# Targeting Nuclear Import of Thyroid Hormone Receptor Alpha with Natural Polyphenols: Potential Mechanism for Lipolysis in Metabolic Dysfunction-Associated Steatotic Liver Disease

**DOI:** 10.3390/cimb48090873

**Published:** 2026-08-28

**Authors:** Evangelia K. Konstantinou, Athanasios A. Panagiotopoulos, Maria Dimitriou, Elias Castanas

**Affiliations:** 1 Department of Nutritional Science and Dietetics, School of Health Sciences, University of the Peloponnese, Antikalamos, 24100 Kalamata, Greece; e.konstantinou@go.uop.gr; 2 Laboratory of Experimental Endocrinology, School of Medicine, University of Crete, Voutes Campus, 71013 Heraklion, Greece; castanas@uoc.gr

**Keywords:** metabolic dysfunction-associated steatotic liver disease (MASLD), thyroid hormone receptor alpha (THRA), nuclear import, importins, polyphenols

## Abstract

Metabolic dysfunction-associated steatotic liver disease (MASLD) is characterized by excessive liver lipids and metabolic imbalance. Thyroid hormone receptor alpha (THRA) is essential for liver lipolysis; however, its transcription function is dependent on successful nuclear localization. Since natural polyphenols are known to modulate THRA activity, making them potential candidates for MASLD management, their roles in THRA nuclear trafficking have not been elucidated. In this study, an integrative computational method was utilized to investigate the impact of polyphenol binding on THRA nuclear import. Protein–protein docking simulations suggested that polyphenol binding may augment the interaction of THRA and importin-α, while importin-7 was predicted to be essential for basal transport. In conclusion, our findings showed that polyphenol binding could potentially support THRA nuclear import, thus potentially implicating a new process in MASLD.

## 1. Introduction

Metabolic dysfunction-associated steatotic liver disease (MASLD) is the most common chronic liver disease and is a major cause of liver-related disorders and heart disease [1,2]. MASLD can develop from simple steatosis to steatohepatitis, fibrosis, cirrhosis, and hepatocellular carcinoma [3,4,5]. It is characterized by excessive lipid accumulation in hepatocytes, accompanied by insulin resistance, obesity, and metabolic dysregulation [6,7]. Despite its rising prevalence, its treatment continues to be limited, making it an area that requires treatment strategies based on underlying mechanistic pathways that selectively target lipid metabolism in the liver [8,9,10].

Thyroid hormones are key players in maintaining energy homeostasis and lipid metabolism, by acting with powerful downstream effects on the hepatic oxidation of fatty acids and removal of triglycerides [11,12,13], mainly mediated by thyroid hormone receptors alpha and beta. Although thyroid hormone receptor beta (THRB) is conventionally regarded as the predominant isoform in adult hepatocytes, emerging evidence demonstrates that thyroid hormone receptor alpha (THRA) plays a distinct, non-redundant role in regulating hepatic fatty acid oxidation, mitochondrial dynamics, and fine-tuning lipolytic gene networks [14,15,16]. THRA acts as a transcription factor that controls the expression of genes related to lipolysis, mitochondrial *β*-oxidation, and lipid export upon ligand binding [14,15,16]. Consequently, ligand-dependent THRA selective activation or its nuclear availability has been suggested as a new and safer method of reducing hepatic lipid accumulation, without the negative consequences of high concentrations of thyroid hormones [14,15,16].

In addition to ligand binding and transcriptional co-factor recruitment, THRA’s intracellular localization is crucial for its genomic activity [17,18]. To exert its classical genomic actions, nuclear THRA typically heterodimerizes with the retinoid X receptor (RXR) at thyroid hormone response elements (TREs) in target gene promoters, driving transcription upon ligand-induced displacement of corepressors and recruitment of coactivators [19]. THRA undergoes regulated cytoplasmic-nuclear shuttling, which is a crucial control point in thyroid hormone signaling [20]. THRA also initiates rapid, extranuclear non-genomic actions; for instance, it physically associates with the p85α subunit of phosphatidylinositol 3-kinase (PI3K) in the cytoplasm, triggering the rapid activation of the PI3K/Protein kinase B (Akt) pathway independently of immediate gene transcription [21]. The import of transcription factors (including nuclear receptor family members) into the nucleus is carried out by the importin-related transport, recognizing nuclear localization signals (NLSs) and mediating crossing of the nuclear membrane, through the nuclear pore complex [22,23,24,25,26]. Importin-α (IPOα) and importin-7 (IPO7) have been implicated in the nuclear transport of (liganded or not) thyroid hormone receptor alpha (THRA), suggesting that ligand binding may influence their nucleocytoplasmic shuttling. Although thyroid hormone receptors are usually characterized as constitutively nuclear, new evidence indicates they conduct active transport, where ligand-induced conformational changes could affect their detection by the nuclear import system [26,27].

Ligand binding has the capability to induce a three-dimensional conformational change in nuclear receptors, especially in their hinge and C-terminal regions, where the ligand recognition site resides [28,29,30,31]. This ligand-induced conformational change subsequently impacts the receptor’s affinity for protein–protein interactions, including its binding with the nuclear transport mechanism [28,29,30,31]. However, the extent to which small molecules regulate THRA nuclear import, through direct effects on importin binding, remains poorly understood [27]. This gap is particularly relevant in the pathophysiology of metabolic dysfunction-associated steatotic liver disease (MASLD), as impaired or suboptimal nuclear import of THRA directly reduces its lipolytic transcriptional output, even in the presence of ligand activation [27].

Natural polyphenols, which are widely distributed in fruits, vegetables, and other dietary sources [32,33,34], are also being sought as metabolic pathway modulators [35,36,37,38] having diverse biological activities [39,40]. The ability of various polyphenolic molecules to interact with THRA, with a mechanism similar to the native ligand triiodothyronine (T3), has already been examined by our group in a previous study, and the results show that these molecules can indeed interact with THRA, supporting the notion that food-derived molecules can act as mild/partial THRA agonists, thus helping to modulate the regulation of hepatic lipid metabolism [34]. Nevertheless, whether such compounds influence downstream events beyond receptor activation, including THRA nuclear trafficking, has not been systematically investigated.

In the present study, we explore a novel mechanistic layer, linking dietary polyphenols to hepatic lipolysis, by focusing on the nuclear import of the receptor. Using a computational model simulation approach, this study evaluates the impact of binding interactions between liganded THRA (with the natural ligand or polyphenols) and the primary nuclear transport acceptor proteins IPOα/IPO7. The study hypothesis is that THRA nuclear uptake could be enhanced through an increased interaction between THRA and importin complexes, introducing a new mechanism in MASLD control, using a pathway where THRA nuclear uptake could control lipolysis gene expression.

## 2. Materials and Methods

Amino-acid sequence for THRA was retrieved from UniProt (https://www.uniprot.org/uniprotkb/P10827, accessed on 10 June 2026). Multiple alignments for each NLS sequence [monopartite Importin-α NLS KRRR and KRKXK [41,42,43,44,45,46,47,48] and (L)PPRS(G/P)P, KP(K/Y)LV, for Importins 4 and 5, respectively [25], EKRKI(E/R)(K/L/R/S/T) for importin-7 [26], and [RRKLPVGRS] for Importin-8 [24]] were identified with the online Clustal Omega tool on the EMBL-EBI server (https://www.ebi.ac.uk/Tools/msa/clustalo/, accessed on 10 June 2026) [49,50]. Only sequences with at least 50% similarity in the amino acids of each NLS were retained. This permissive 50% cutoff was selected as an exploratory screening threshold to capture potential non-canonical or degenerate NLS patterns thatmight otherwise be missed by strict consensus filtering. Through this approach, target interface regions were identified on THRA, including the predicted IPOα binding region at residues 20–30 (^20^RSPDGKRKRKN^30^) and the IPO7 binding region at residues 130–150 (^130^KRVAKRKLIEQNRERRRKEEM^150^). The NLS region alignment was constructed, analyzed and visualized in JalView v2.11.1.7 [51].

The three-dimensional structure of THRA was retrieved from the AlphaFold Protein Structure Database (https://alphafold.ebi.ac.uk/, accessed on 15 September 2025) [52] (Code: AF-P10827-F1) in PDB format. Polyphenolic compounds and endogenous agonist T3 were obtained from the PubChem database (https://pubchem.ncbi.nlm.nih.gov/, accessed on 15 September 2025) [53] in canonical SMILES format and converted to PDB files using Open Babel (http://openbabel.org, accessed on 15 September 2025) [54]. Specific polyphenols were selected based on our previous screening and docking analysis [34], which identified them as promising candidates for binding to THRA. The procedure followed for the preparation of THRA, flexible binding of ligands, and hydration of complexes was described in our previous work [34].

The three-dimensional structures of importin-α/importin-β/Ran-GDP (IPOα/IPOβ/Ran-GDP) and importin-7/Ran-GDP (IPO7/Ran-GDP) complexes were generated and prepared in PDB format for protein–protein docking using the in silico methodology described in reference [26]. Structural integrity and orientation were verified using UCSF Chimera software (https://www.cgl.ucsf.edu/chimera/, accessed on 10 June 2026) [55] prior to docking simulations.

Protein–protein docking simulations were carried out using the HEX 8.0.0 software (http://hex.loria.fr/, accessed on 10 June 2026) [56]. Both unliganded and ligand-bound THRA conformations were docked independently with IPOα/IPOβ/Ran-GDP and IPO7/Ran-GDP to simulate the physiological assembly of the nuclear import complex within the cytoplasm and to evaluate the effect of ligand-induced conformational changes on importin binding. Prior to docking, all structural models were prepared by adding missing hydrogen atoms and assigning standard protonation states at physiological pH 7.4. Docking calculations were executed using a 3D Fast Fourier Transform (FFT) correlation algorithm based on Shape + Electrostatics mode. Sampling was performed using the Range Angles method with full rotational coverage (Receptor Range: 180°, Step Size: 7.5°; Ligand Range: 180°, Step Size: 7.5°; Twist Range: 360°, Step Size: 5.5°). The translational search parameters were set to a Distance Range of 40 Å, Box Size of 10 Å, Translation Step of 0.8 Å, and Grid Dimension of 0.6. For each simulation, 2000 docking solutions were generated and ranked according to their HEX docking energy scores (E_total_). Top-ranked pose clusters were selected for conformational and downstream binding interface analysis.

Comparative structural analyses between unliganded THRA-importin complexes and ligand-bound THRA-importin complexes were performed using PyMOL V2.4 (https://www.schrodinger.com/, accessed on 12 June 2026) and UCSF Chimera software (https://www.cgl.ucsf.edu/chimera/, accessed on 12 June 2026). Special attention was paid to detecting conformational changes occurring within THRA regions that were associated with nuclear localization signal regions (NLS regions), such as surface changes and interfacial interactions with importins. The possibility of a conformational alteration of THRA favorable for importin binding or recognition was systematically investigated, based on binding orientation, binding interfaces, and binding energies. Possible relationships between conformational changes in THRA and its improved nuclear import ability were deduced.

## 3. Results

Several conserved NLS motifs that may be involved in several importin-dependent nuclear import pathways were found through sequence-based analysis of THRA. Multiple sequence alignment revealed specific regions in THRA’s hinge region and N-terminal domain that strongly resemble well-characterized monopartite and bipartite NLS motifs for IPOα binding [41,42,43,44,45,46,47,48]. In addition, certain sequence features were consistent with transport mediated by IPO7 [26]. Particularly, a monopartite IPOα-associated NLS was correlated with a cluster of residues between amino acids 20–30. A second region, which covered amino acids 130–150, overlapped with the previously described NLS element recognized by IPO7 [26]. These regions and importin complexes were therefore selected for subsequent structural modeling and protein–protein docking studies, as they likely represent key interfaces involved in the importin-mediated nuclear transport of THRA.

Sequence alignment analysis revealed high similarity scores exceeding the 50% threshold within these identified regions. Specifically, for the predicted IPOα binding region (residues 20–30, ^20^RSPDGKRKRKN^30^), the basic core motif ^25^KRKRK^29^ demonstrated 100% identity with the canonical monopartite importin-α consensus motifs (KRRR and KRKXK). Similarly, for the predicted IPO7 binding region (residues 130–150, ^130^KRVAKRKLIEQNRERRRKEEM^150^), the embedded sequence ^134^AKRKLIE^140^ exhibited 70% similarity to the reference importin-7 motif (EKRKI(E/R)(K/L/R/S/T)). Additionally, an analysis was performed and no match was found with other known NLSs that recognize other importins (4, 5, and 8).

**Figure 1:**
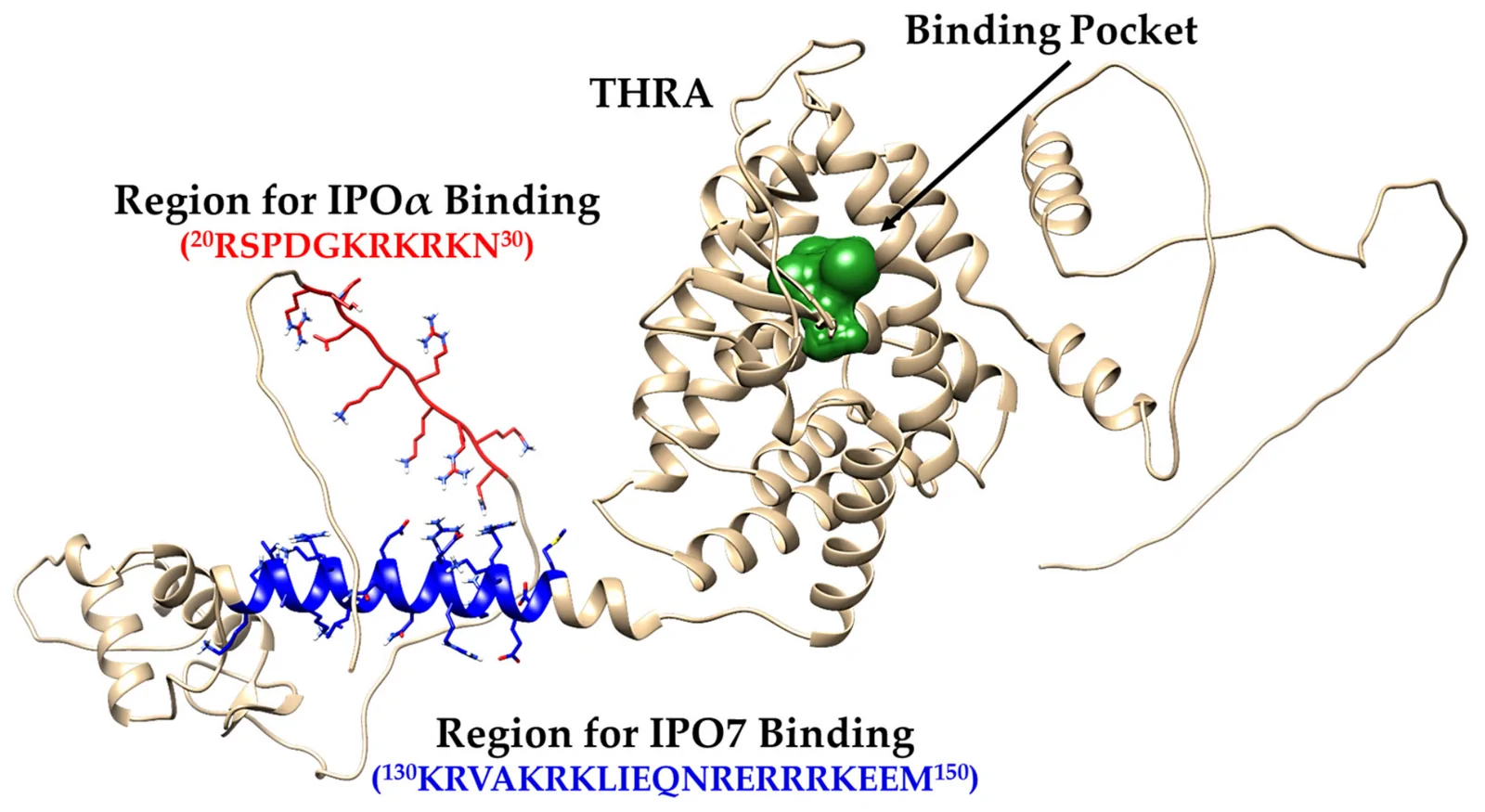
Structural representation of THRA highlighting the ligand-binding pocket and the predicted importin interaction regions. The three-dimensional structure of THRA was obtained from the AlphaFold Protein Structure Database (https://alphafold.ebi.ac.uk/, accessed on 15 September 2025) [52] (Code: AF-P10827-F1) and visualized using UCSF Chimera software (https://www.cgl.ucsf.edu/chimera/, accessed on 12 June 2026) [55]. The ligand-binding pocket is indicated by the green shape, while the predicted binding regions involved in interactions with IPOα and IPO7 are shown in red and blue, respectively.

Structural mapping of the identified NLS domains in the THRA three-dimensional structure revealed a clear separation between the ligand binding pocket and the predicted importin interaction interfaces. As shown in Figure 1, the ligand binding pocket is located in a different region from the regions involved in interactions with both IPOα and IPO7. The predicted IPOα interaction site was located mainly within the N-terminal domain, spanning amino acids 20–30, while the IPO7 interaction interface was mapped to the hinge region spanning amino acids 130–150. This distinctive spatial arrangement raises the question of whether and how ligand binding in the LBD may differentially influence these transport interfaces. In particular, although the flexible nature of the N-terminal IPOα site may be affected by allosteric shifts, it remains to be determined if the structured hinge region which includes the IPO7 site undergoes similar ligand-induced orientation changes. The ligand binding pocket is physically separated from the NLS motifs but binding of the ligand to the Ligand Binding Domain (LBD) triggers long-range conformational changes that modulate the exposure of these transport signals. Furthermore, the location of the IPOα-NLS within a flexible, non-structured region renders its accessibility highly sensitive to these allosteric changes, modulating its binding dynamics with importins.

**Figure 2:**
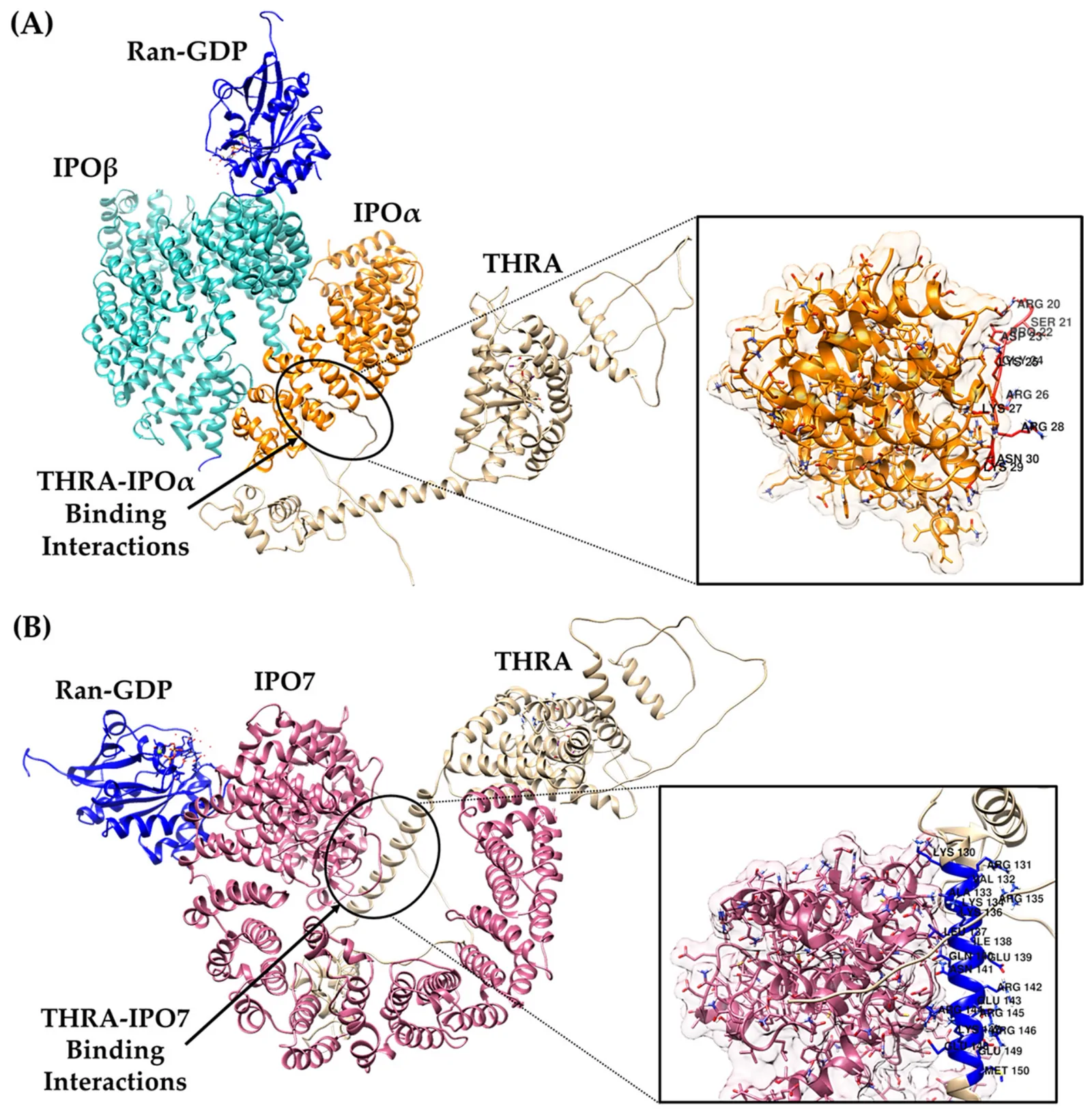
Protein–protein docking models illustrating the interaction of THRA with importin complexes. The three-dimensional structures of THRA and importins were obtained from the AlphaFold Protein Structure Database (https://alphafold.ebi.ac.uk/, accessed on 15 September 2025) [52], while protein–protein docking was performed using HEX 8.0.0 software (http://hex.loria.fr/, accessed on 10 June 2026) [56]. Structural visualization and analysis were carried out using UCSF Chimera software (https://www.cgl.ucsf.edu/chimera/, accessed on 12 June 2026) [55]. (**A**) Docking model of THRA interaction with the IPOα/IPOβ/Ran-GDP complex. (**B**) Docking model of THRA interaction with the IPO7/Ran-GDP complex. In both panels, THRA is shown in gold, IPOα in orange, IPO7 in pink, IPOβ in light blue, and Ran-GDP in blue. Insets in each panel highlight magnified views of the interaction interfaces, where THRA amino acid residues involved in binding to IPOα are shown in red and those interacting with IPO7 are shown in blue.

Protein–protein docking simulations were performed to assess the interactions between both unliganded and ligand-bound THRA conformations and the IPOα/IPOβ/Ran-GDP and IPO7/Ran-GDP complexes. Representative docking models are shown in Figure 2, which depict distinct binding orientations of THRA with each importin complex. The docking analyses showed that THRA interacts with IPOα and IPO7 through partially overlapping but structurally distinct regions, consistent with predictions from sequence-based NLS analysis and previous work that has identified cargo protein binding sites on IPOα (Arm2-Arm4 domains, AA 121–247) and IPO7 (AA 602–751) [26]. Notably, ligand-bound THRA conformations exhibited altered binding geometries compared to the unliganded one, suggesting that ligand binding modifies the conformation of the THRA-Importin interaction interface.

The quantitative analysis of THRA interactions with IPOα is summarized in Table 1. The protein–protein binding results showed that ligand-bound THRA complexes exhibited strong binding to IPOα as reflected by the large docking binding scores, in contrast to the unliganded receptor, which was found not to bind to IPOα. Structural superposition of ligand-bound THRA-IPOα complexes with the reference T3-bound conformation revealed distinct differences within the N-terminal binding interface (amino acids 20–30), as reflected in the Root-Mean-Square Deviation (RMSD, Å) measurements. Comparative visualization of this region revealed ligand-specific rearrangements of THRA residues involved in IPOα recognition, suggesting that ligand binding modifies the structural conformation of the IPOα interaction surface.

The docking analysis of THRA interactions with IPO7 is summarized in Table 2. Consistent with the observations for IPOα, ligand-bound THRA conformations exhibited altered predicted binding scores and interaction interfaces compared with the unliganded receptor. RMSD analysis focused on the hinge region (amino acids 130–150) revealed receptor-dependent deviations from the reference THRA conformation bound to T3. Structural superpositions indicated that receptor binding induces conformational adaptations within this region, which may influence the interaction of THRA with IPO7. These findings suggest that distinct ligands differentially modulate THRA recognition in alternative nuclear entry pathways. It is noteworthy that unliganded THRA, unlike IPOα, can bind to IPO7.

These data suggest that the ligand-binding domain (LBD) is predicted to allosterically communicate with distant parts of the receptor, including the remote N-terminal domain. Our models indicate a plausible dual mechanism, where unliganded THRA exhibited low predicted compatibility for IPOα within our computational workflow but has a high predicted interaction potential for IPO7 (E_total_ = −3756.61). However, both pathways are affected when natural polyphenols or T3 are binding to LBD. Polyphenol or T3 binding may act as a potential molecular switch for the flexible N-terminus (AA 20–30) inducing tight binding to IPOα (E_total_ down to −1990.02) and major structural changes (RMSD up to 12.670 Å). Moreover, in the structured hinge region (AA 130–150), both T3 and natural polyphenols modulate and fine-tune the interaction with IPO7, with some compounds predicted to form tighter complex formations, as indicated by more favorable interaction energy scores (down to −5171.51) and change its conformation (RMSD up to 2.919 Å). The present data finally suggest that ligand binding, whether by endogenous hormones or dietary polyphenols, modifies and increases the interactions of THRA with alternative importin pathways, which may structurally favor its importin-mediated nuclear transport.

## 4. Discussion

THRA plays a crucial role as a key regulatory element in the lipid metabolism of the liver and must maintain intact functionality in order to avoid excessive lipid accumulation within hepatocytes [13,58,59,60]. Although the activation of the ligand-functionalized form of THRA has been investigated thoroughly in the literature, the regulatory processes associated with the intracellular transport and its availability in the nucleus have been less well studied [61,62]. As a member of the transcription factor family, the functional efficacy of THRA in the mediation of lipolytic responses is ultimately associated with successful transport into the cell nucleus, binding in an unliganded form to specific DNA elements in heterodimeric form with RXR and ligation to available T3 [20]. In this context, it is important to note that T3 is conventionally recognized for its principal transcriptional functions within the nucleus, but increasing evidence indicates its capacity to bind and interact with signaling proteins also in the cytosol [63,64]. This cytosolic binding of T3 is known to activate rapid, non-genomic signaling pathways (e.g., Mitogen-activated protein kinase (MAPK) or PI3K/Akt cascades) and can actively influence thyroid hormone receptor trafficking dynamics [63,64]. Therefore, the presence of these cytoplasmic interactions adds further biological relevance to our model, indicating that the receptor can indeed interact and bind to ligands, whether they are endogenous hormones or dietary polyphenols, in the cytoplasm prior to importin-mediated nuclear translocation. This problem is addressed in the current study using the in silico approach in order to show that the levels of natural polyphenolic compounds could potentially influence the regulatory processes associated with the transport of THRA into the nucleus.

The results from our research suggest that there are various conserved NLS sequences found mostly in the N-terminal region and hinge regions of the THRA protein. Interestingly, the structural landscape of these NLS motifs is dramatically different. IPOα-NLS is located in a non-structured, highly flexible loop (highlighted in red in Figure 1), suggesting an ‘induced fit’ mechanism upon binding, allowing fast access to the nuclear import machinery. For other pathways, the potential NLS regions are located in more rigid structured domains. This unique structural organization suggests that the non-structured NLS may be recognized constitutively or with high sensitivity and that the structured NLS domains are likely to require ligand-induced allosteric conformational changes to become exposed and functional. The degree of sequence similarity of these regions to known recognition sites for IPOα and IPO7 suggests that more than one nuclear import pathway is employed by the THRA protein [26,27]. The distribution of these NLS regions on the 3D structure of the THRA protein shows that they do not lie within or in the proximity of the ligand-binding pocket. This observation implies that ligand binding does not directly mask or expose the NLS motifs through steric interactions but rather induces long-range conformational changes that alter the accessibility or orientation of importin recognition surfaces. Our computational data reveal distinct energetic profiles between the two states, with the unliganded receptor showing a different mode of interaction compared to the liganded complex.

Protein–protein docking simulations further clarified the functional implications of these structural changes. In the ligand-unbound state, THRA was predicted to strongly interact with IPO7 but was unable to constitute a stable complex with IPOα. This result is consistent with experimental data pointing to IPO7 as a basal nuclear importer of THRA and arguing for a potential IPO7-import mediated constitutive pathway for maintaining a certain level of cellular nuclear receptor presence under basal conditions [27,65]. In sharp contrast, ligand-bound THRA structural models, including complexes with natural polyphenols as well as with the endogenous ligand T3, strongly interacted with the IPOα/IPOβ/Ran-GDP complex. These data indicate that activation of the receptors leads to an extension of the recognition capabilities of THRA for IPOα, which is predicted to favor import efficiency.

Crucially, the observed enhancement in IPOα binding was seen to occur in a manner that is coupled with ligand-specific structural changes in the N-terminus of the THRA protein, specifically in the 20–30 amino acid stretch that was revealed to be a prime binding interface for IPOα. Root mean square distance analysis further indicated that the structural changes in the 20–30 amino acid stretch, as well as in the 130–150 amino acid stretch in the “hinge” domain that mediates binding with IPO7, were ligand-specific, suggesting that importin recognition dynamics may be sensitive to the nature of the bound ligands that are specifically bound to the receptor [26,27,65].

From a biological perspective, the impact of THRA nuclear import could have highly significant effects on liver lipid metabolism [66]. While THRΒ remains the primary target for systemic hepatic thyroid signaling, our findings highlight the THRA-importin axis as a subtle, non-redundant regulatory node. A moderate augmentation in the amount of THRA in the nucleus could significantly enhance the transcription of genes involved in β-oxidation in mitochondria, lipid transport, and triglyceride uptake [14]. In MASLD, in which the efficiency of signaling rather than the complete absence of the functionally affected receptor plays a prominent part in the pathogenesis, an augmentation in nuclear import via IPOα/ΙPO7 could be an efficacious method of intervention by optimizing promoter occupancy at lipolytic gene loci [14]. The effect of polyphenols on nuclear import induction could therefore hypothetically support lipolytic pathways by increasing the receptor availability in the nucleus. This increase in nuclear presence is a prerequisite for the function of the THRA but the biological effect will depend on the ability of the receptor to engage with DNA (primarily as an RXR heterodimer at TREs) and to initiate transcription when bound to these compounds that remains to be further investigated. However, this mechanism may offer a means of sensitizing cells to metabolic signaling, potentially reducing the need for high-dose thyromimetic agents and the risk of systemic side effects [34]. The current observations also provide a mechanistic insight into the pleiotropic metabolic effects that can be anticipated with polyphenol-containing dietary regimens [67,68,69,70]. Although it is established that polyphenols exert strong antioxidant and anti-inflammatory effects, their ability to modulate nuclear receptor trafficking proposes an interesting working hypothesis for a hitherto unrecognized mode of action [71]. Acting via mild/partial THRA ligand activity that concurrently modulates nuclear trafficking, polyphenols could assume interesting functions as fine-tune modulators of metabolic signaling pathways rather than classical high-affinity modulators/agonists [34]. Their dual functions could be particularly welcome in chronic metabolic diseases such as MASLD, where mild metabolic reprogramming is clearly preferable to strong pharmacological manipulation [34].

Another aspect of this research has to do with the differential use of importin routes by the different ligand-bound conformations of THRA. Our data suggest that both import pathways can be utilized, but IPO7 is the preferential route for nuclear translocation of THRA due to its significantly higher binding affinities. Alternatively, ligand binding initiates the IPOα pathway as a secondary or cooperative mechanism. It might be a complex system of fine-tuning nuclear entry. Although these ideas are purely speculative at this point, they highlight the nuances of nuclear receptor regulation and the relevance of considering nucleocytoplasmic transport as a part of the total process of hormone signaling.

Another important aspect regarding the molecular interaction mechanics is whether the above dietary polyphenols have the same binding cavity as the endogenous ligand T3. In our previous work [34], we showed that these specific polyphenolic compounds bind to the same canonical hydrophobic pocket of the LBD of THRA with high binding affinities. This confirmed a competitive binding mode with T3, with a well-established spatial overlap. Moreover, our previous functional characterization [34] has shown that these polyphenols are mild/partial agonists. The current study thus extends these findings to the question of how this competitive binding and ensuing allosteric activation are manifested in terms of regulating the nucleocytoplasmic trafficking dynamics.

However, a number of questions remain unanswered: First, this experiment utilizes the predictions of models and simulations of ligand docking, which do not completely reflect the cellular environment. Although the stability and binding energetics of the ligands within the receptor were verified using MM/GBSA in our previous study [34], and HEX explicitly evaluates 3D surface shape overlap and electrostatic correlation across the protein–protein interface, future Molecular Dynamics (MD) simulations will further clarify the dynamic conformational trajectories of these macromolecular complexes. Furthermore, the bioavailability of natural polyphenols could pose a limitation on their maximum potential concentration to affect the THRA nuclear import function. Additionally, it remains unknown whether polyphenol-bound THRA can effectively bind to DNA and initiate transcription, or how the receptor might switch from polyphenol binding to its endogenous ligand T3 inside the nucleus. Therefore, a number of experimental validation strategies for testing the effects on THRA nuclear import, DNA binding capacity, and functional activity through either importin inhibitors/knockdown or animal models of metabolic liver diseases will have to be employed for confirmation of this hypothesis.

## 5. Conclusions

This study provides the first in silico evidence that natural polyphenols could modulate the activity of THRA through nuclear import, thus broadening our knowledge and interpretation associated with the signaling pathways and action mechanisms initiated by thyroid hormone receptors in liver metabolism. By illustrating and describing how importin is associated with specific ligand-induced structural changes to mediate import processes for triggering signaling pathways within the nucleus, this study proposes a plausible molecular hypothesis through which natural compounds could potentially influence THRA nuclear availability, providing a framework for future in vitro and in vivo studies on metabolic regulation in MASLD.

## Figures and Tables

**Table 1:** Binding affinities, structural comparison, and binding interface superposition of ligand-bound and unliganded THRA complexes with IPOα. The table presents (1) the investigated ligand, (2) its chemical structure, (3) the HEX docking energy score (E_total_) of THRA (ligand-bound or unliganded) to IPOα as calculated by protein–protein docking, (4) the Root-Mean-Square Deviation (RMSD, Å) of the THRA binding region to IPOα compared to the T3-bound THRA complex, and (5) a comparative structural visualization of the THRA binding region to IPOα (amino acids 20–30), where the unliganded THRA is shown in white, ligand-bound THRA is shown in yellow and the T3-bound THRA conformation is shown in red, all in ball-and-stick format. * P1–P4 correspond to natural products selected from the work of Allam et al. in 2020 [57]. It should be noted that, as reported in a previous work, natural polyphenols bind to the ligand-binding pocket of the THRA [34].

Ligand	Structure of Ligand	E_total_ of THRA (with or Without Ligand) Binding to IPOα	RMSD (Å) οf THRA Binding Region to IPOα (with or Without Ligand vs. T3)	Comparative Visualization οf THRA Binding Region to IPOα (AA 20–30) (with or Without Ligand vs. T3)
Without Ligand	-	-	2.082	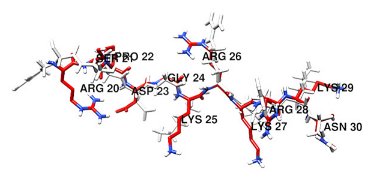
Τ3	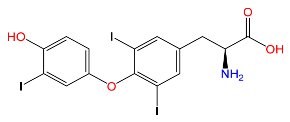	−1493.24	-	-
2-Hydroxycinnamate	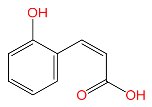	−1990.02	7.833	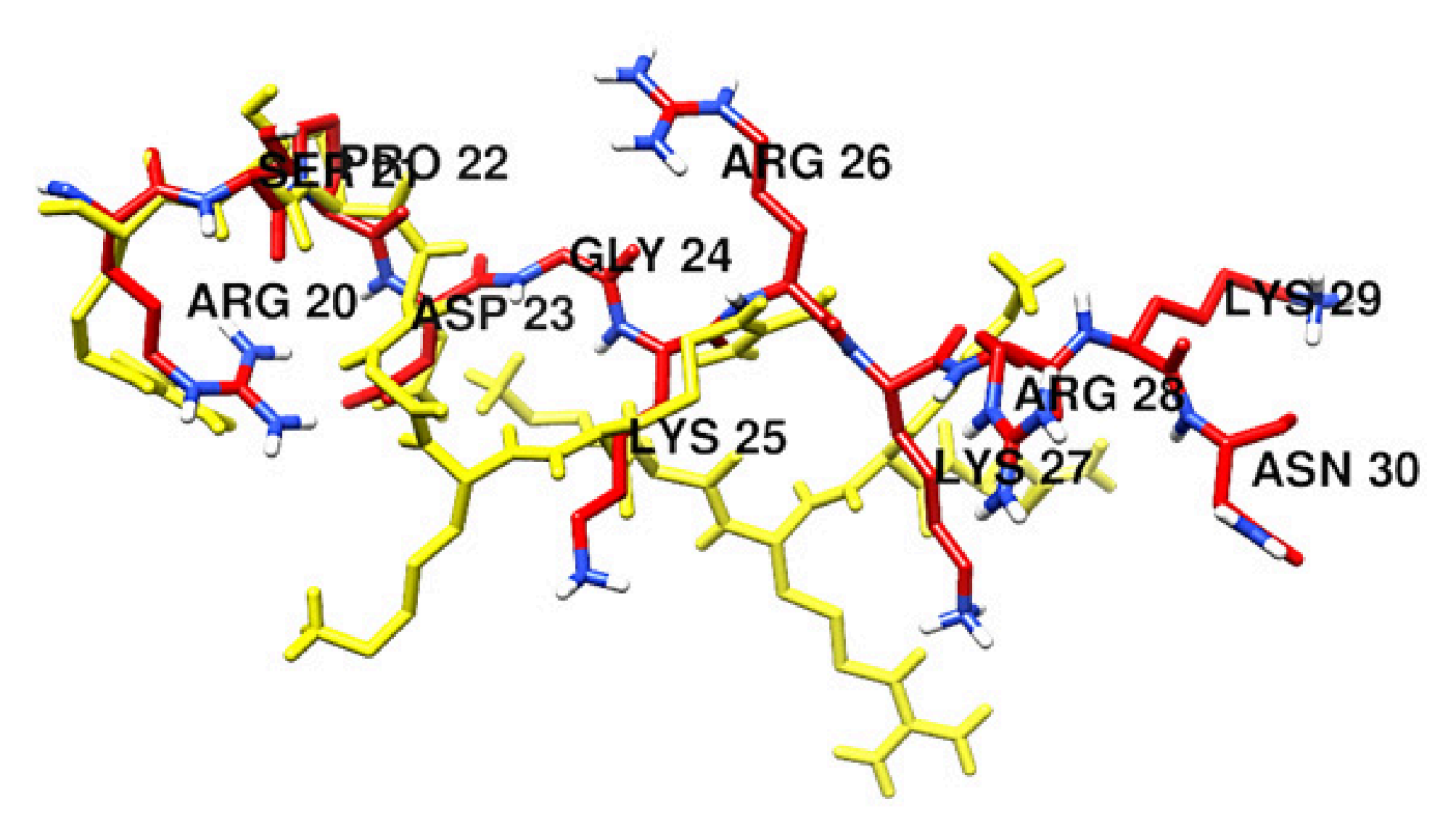
3-Hydroxyflavone	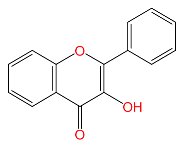	−1055.15	2.385	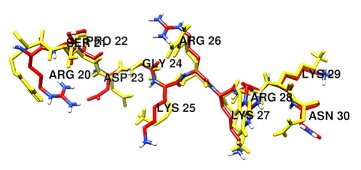
Aurantinidin	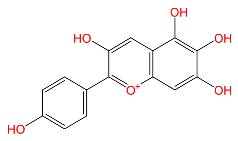	−1682.52	3.313	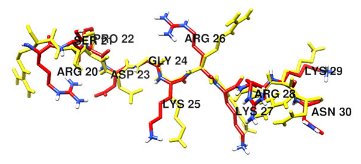
Caffeic Acid	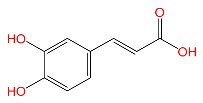	−1792.24	2.549	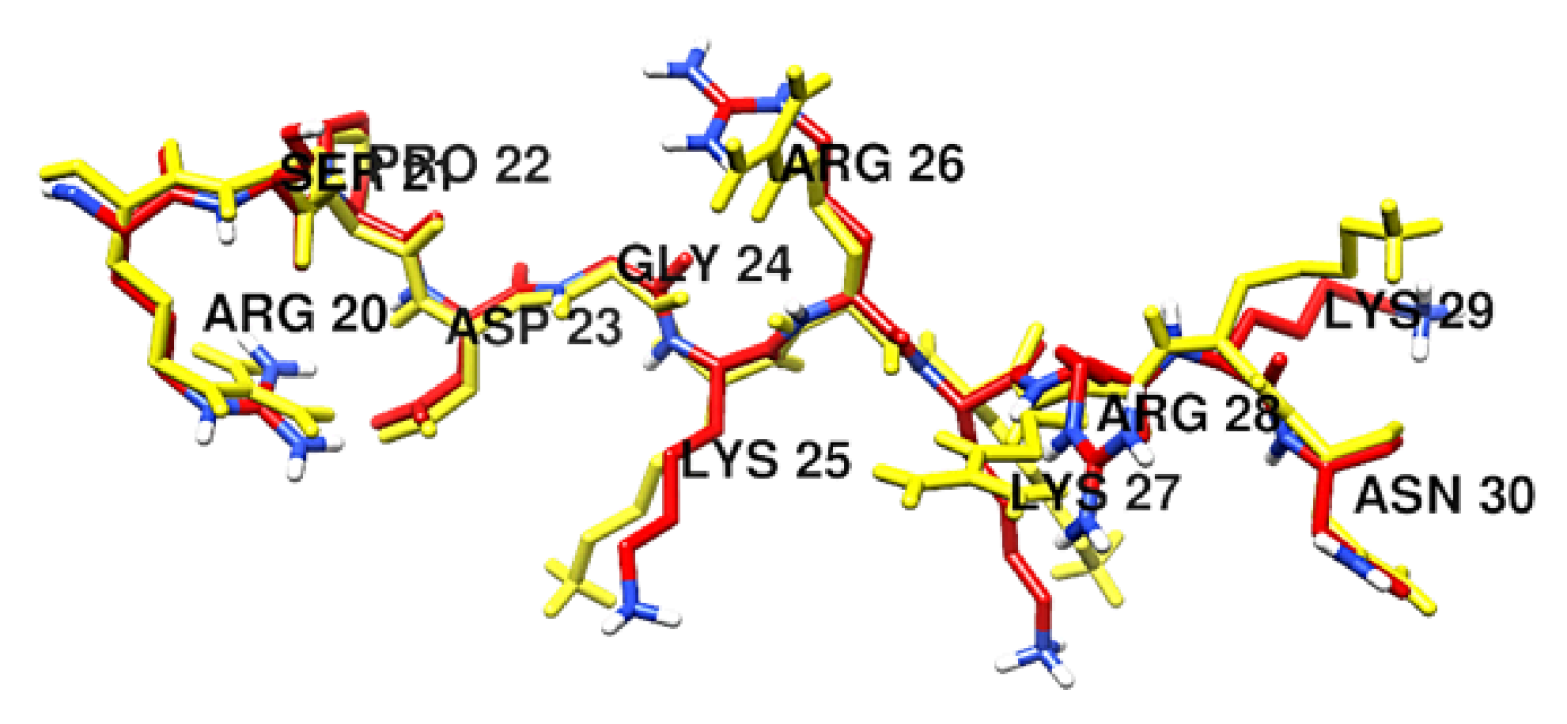
Caffeic Acid Phenethyl Ester	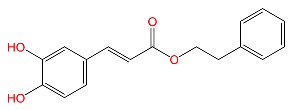	−1252.48	4.055	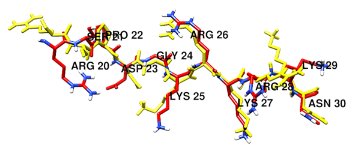
Chlorogenic Acid	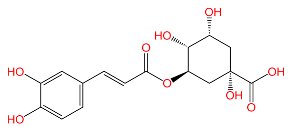	−1637.36	12.670	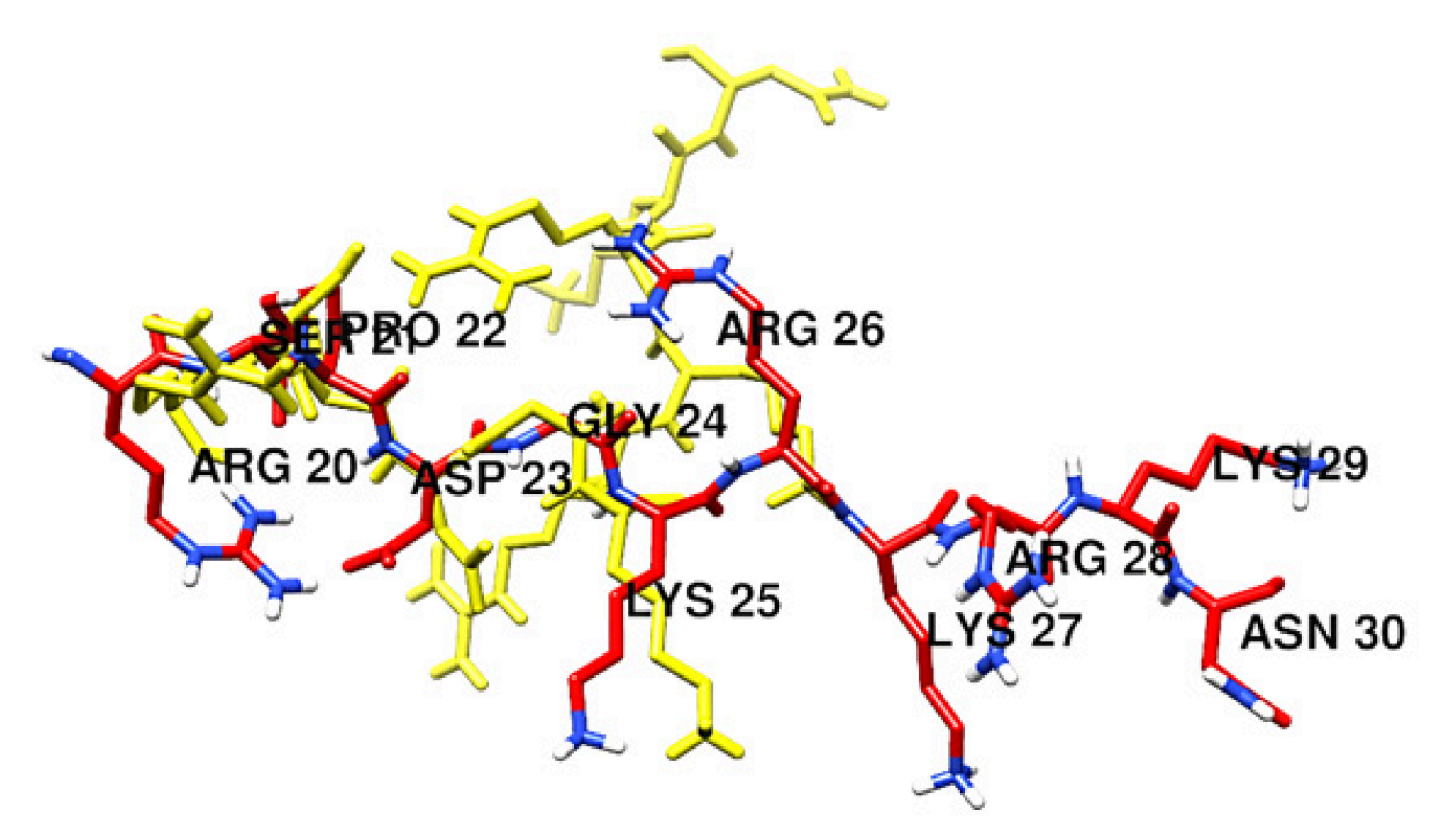
Chlorogenic Acid Methyl Ester	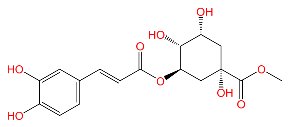	−1668.67	4.025	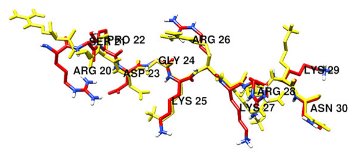
p-Coumaric Acid	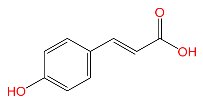	−1350.71	8.328	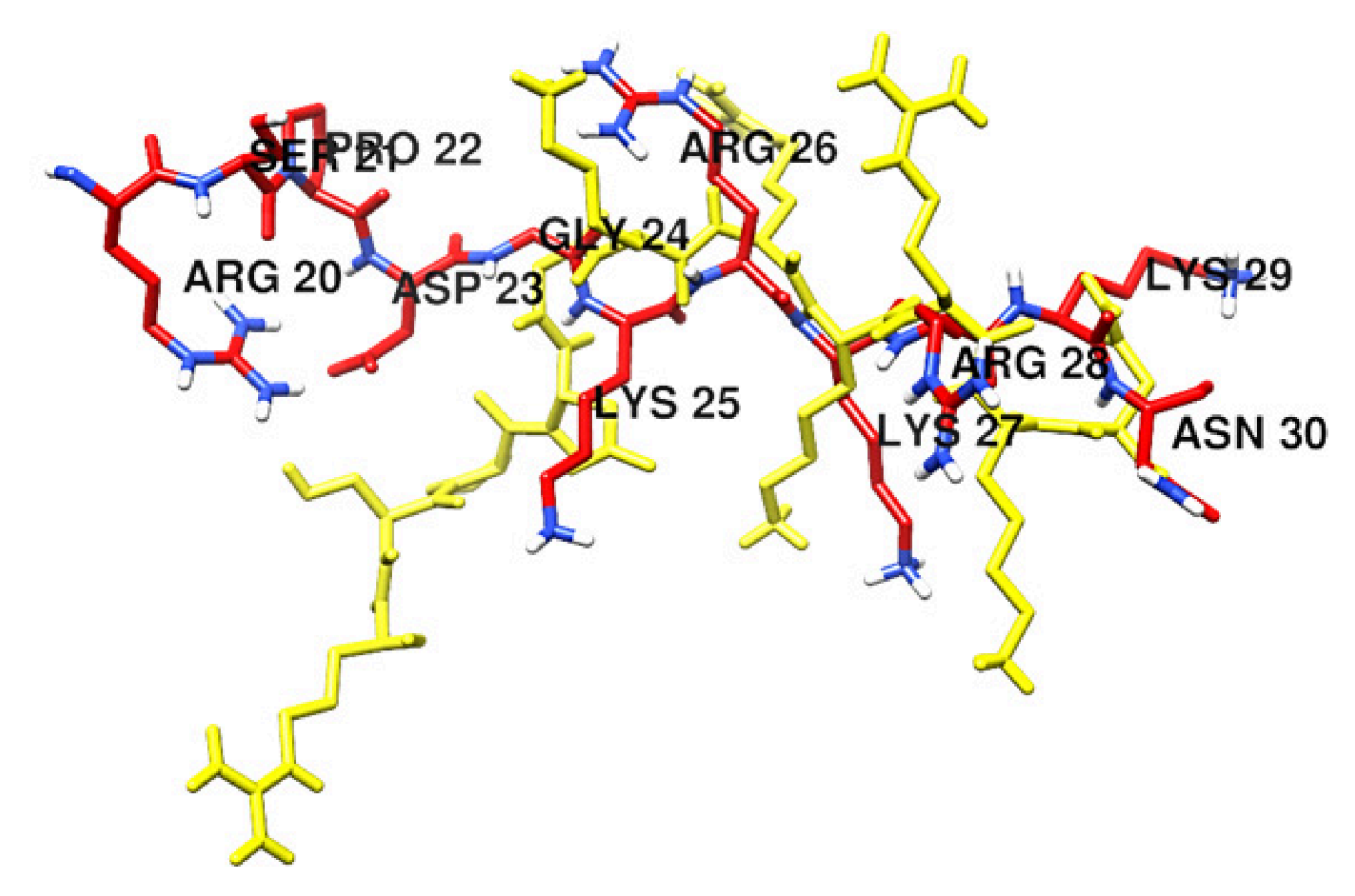
Curcumin	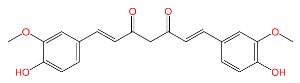	−1430.79	9.040	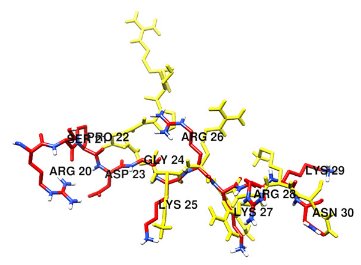
Epicatechin	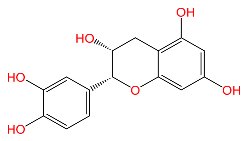	−1550.68	4.205	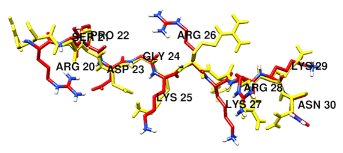
Ferulic Acid	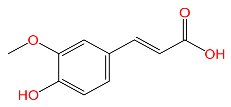	−1409.89	3.881	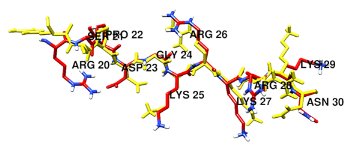
Genistein	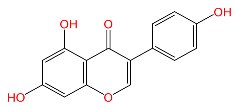	−1645.51	3.520	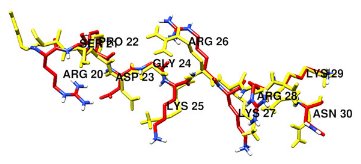
Methyl-4-Hydroxycinnamate	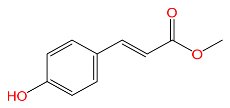	−1543.52	3.604	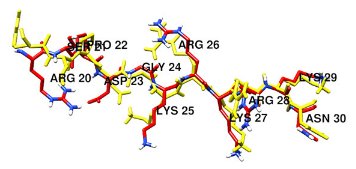
O-Methylsinapate	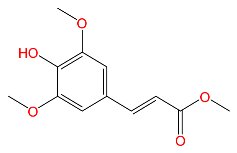	−1681.87	3.569	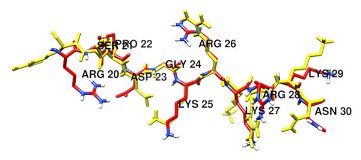
P1 *	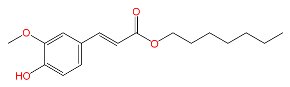	−1687.36	8.201	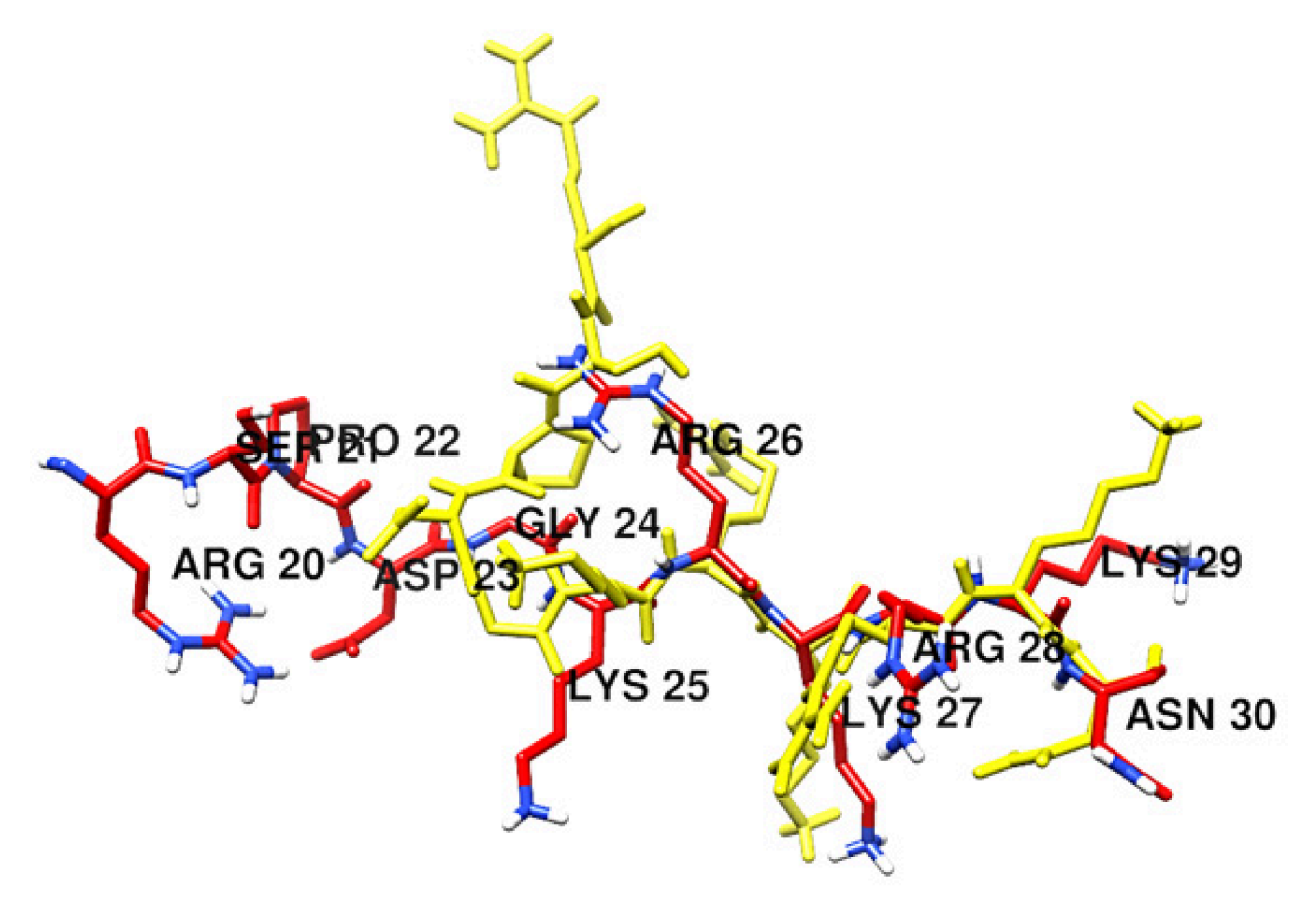
P2 *	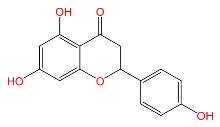	−1398.79	6.520	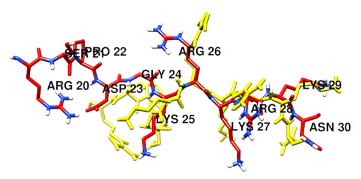
P3 *	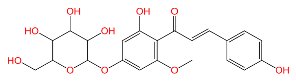	−1649.75	5.791	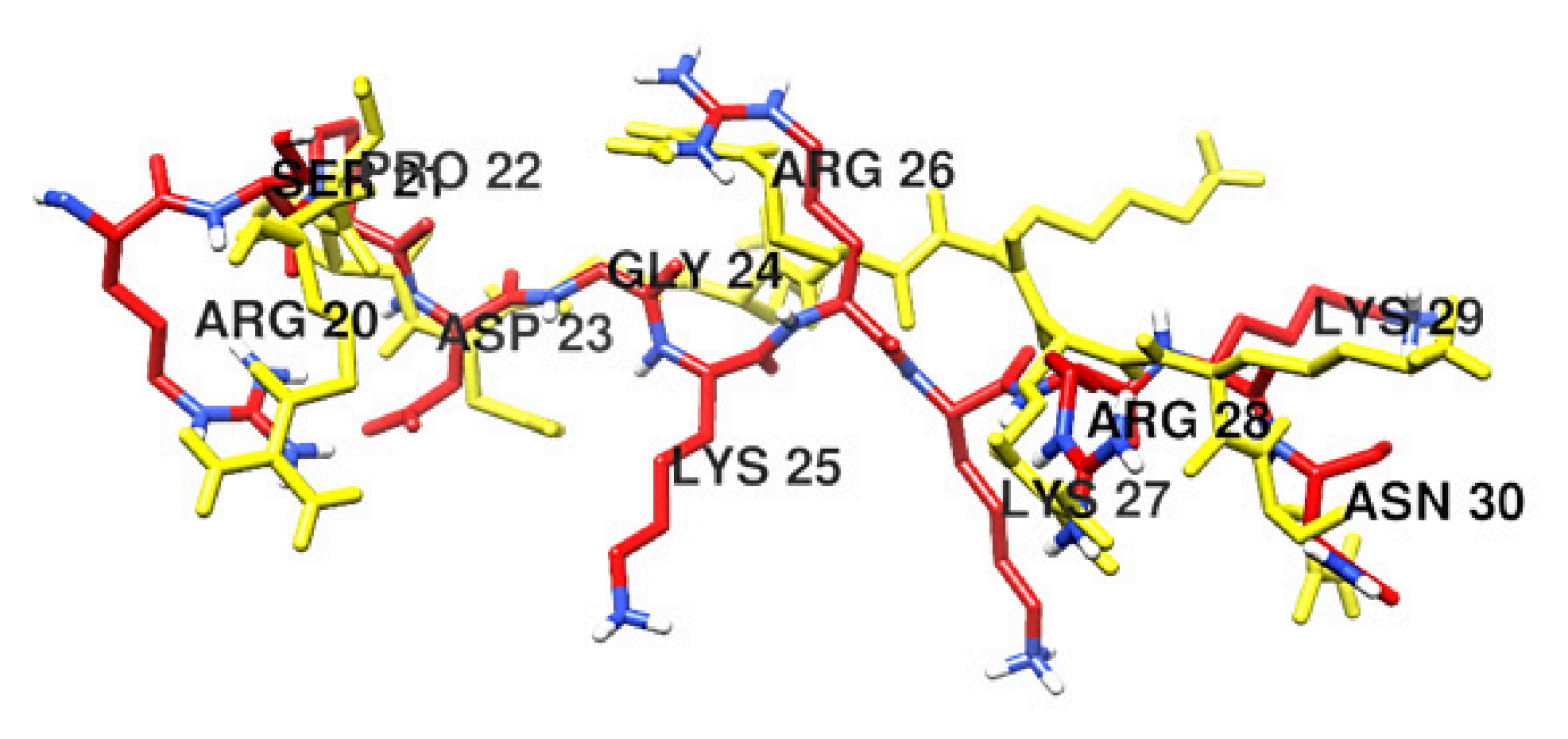
P4 *	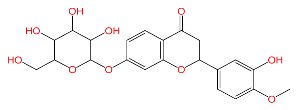	−1800.03	8.977	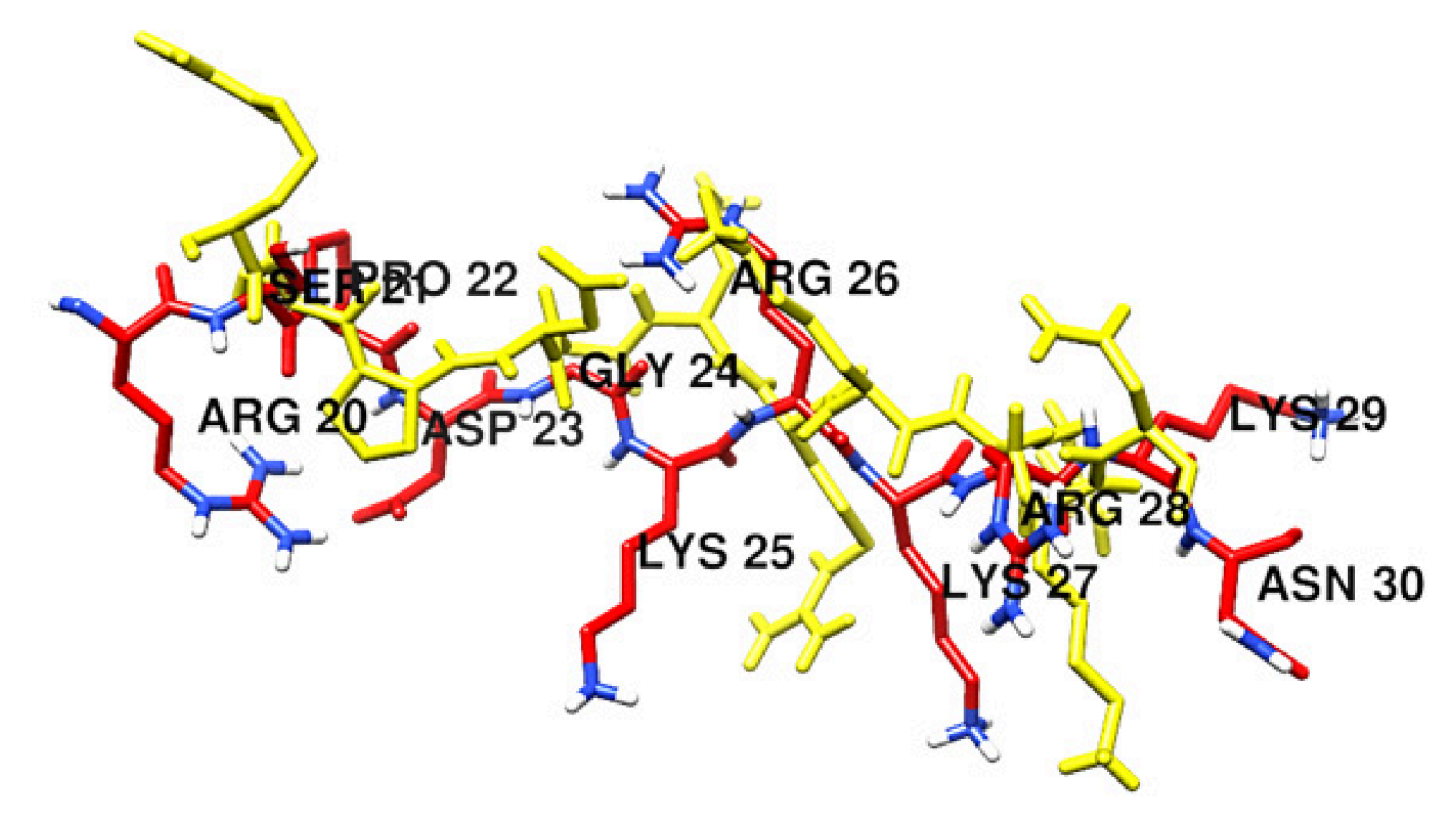
Quercetin-3-Galactoside	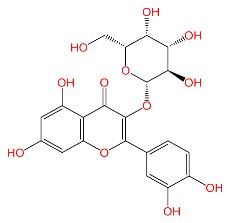	−1462.81	3.721	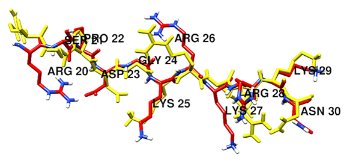
Quercetin-3-Glucoside	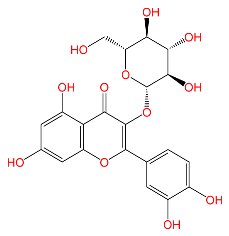	−1740.69	4.392	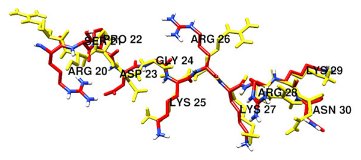
Sinapic Acid	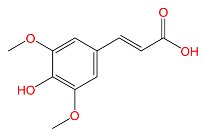	−1447.82	4.243	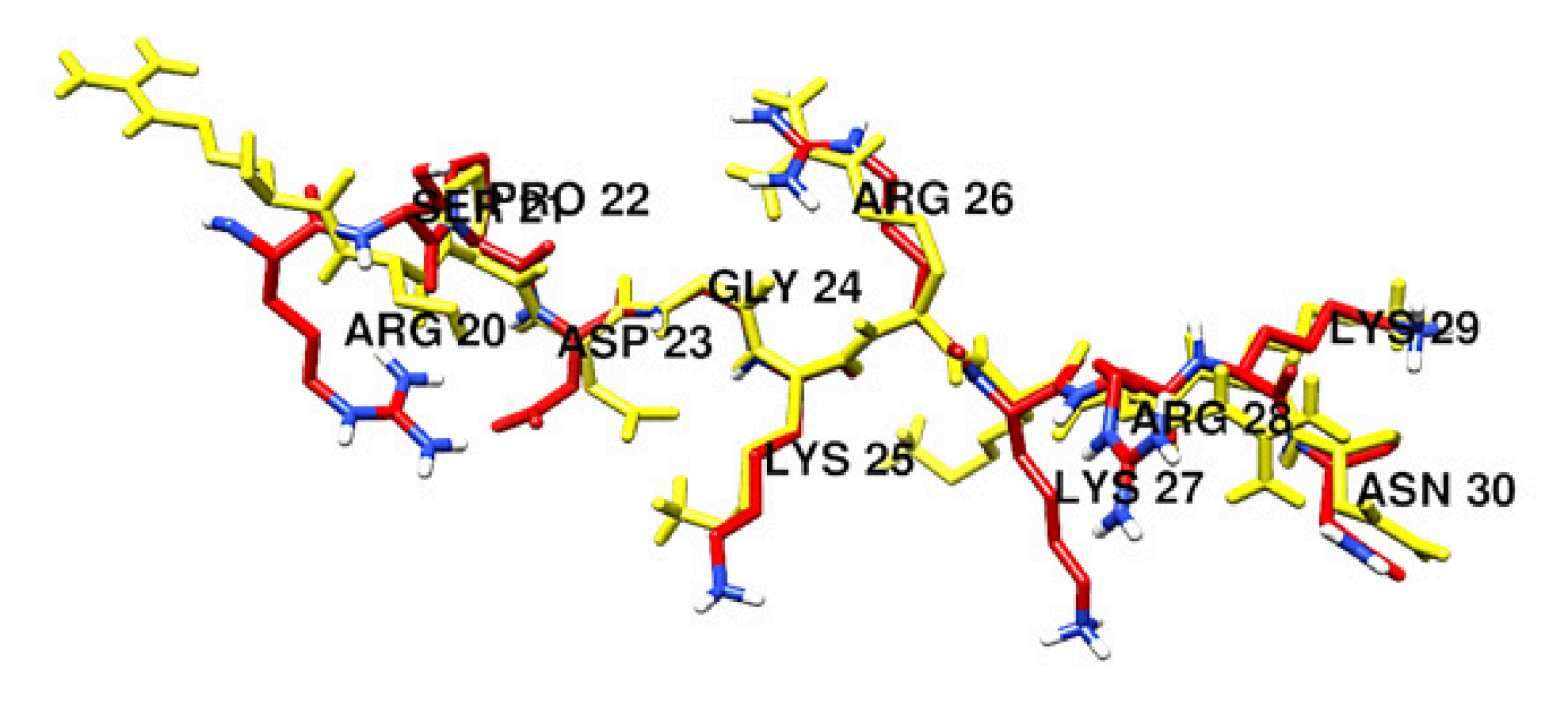

**Table 2:** Binding affinities, structural comparison, and binding interface superposition of ligand-bound and unliganded THRA complexes with IPO7. The table presents (1) the investigated ligand, (2) its chemical structure, (3) the HEX docking energy score (E_total_) of THRA (ligand-bound or unliganded) to IPO7 as calculated by protein–protein docking, (4) the Root-Mean-Square Deviation (RMSD, Å) of the THRA binding region to IPO7 compared to the T3-bound THRA complex, and (5) a comparative structural visualization of the THRA binding region to IPO7 (amino acids 130–150), where the unliganded THRA is shown in white, ligand-bound THRA is shown in yellow and the T3-bound THRA conformation is shown in blue, all in ball-and-stick format. * P1–P4 correspond to natural products selected from the work of Allam et al. in 2020 [57]. It is to note that, as reported in a previous work, natural polyphenols bind to the ligand-binding pocket of the THRA [34].

Ligand	Structure of Ligand	E_total_ of THRA (with or Without Ligand) Binding to IPO7	RMSD (Å) οf THRA Binding Region to IPO7 (with or Without Ligand vs. T3)	Comparative Visualization οf THRA Binding Region to IPO7 (AA 130–150) (with or without Ligand vs. T3)
Without Ligand	-	−3756.61	1.136	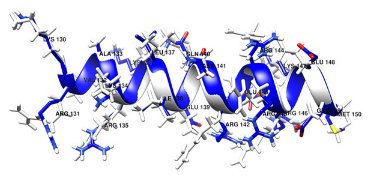
Τ3	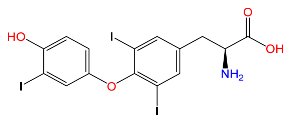	−4219.46	-	-
2-Hydroxycinnamate	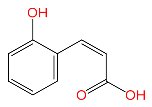	−5094.81	1.895	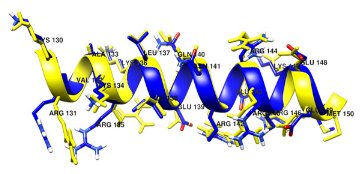
3-Hydroxyflavone	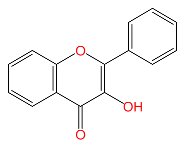	−3303.18	2.025	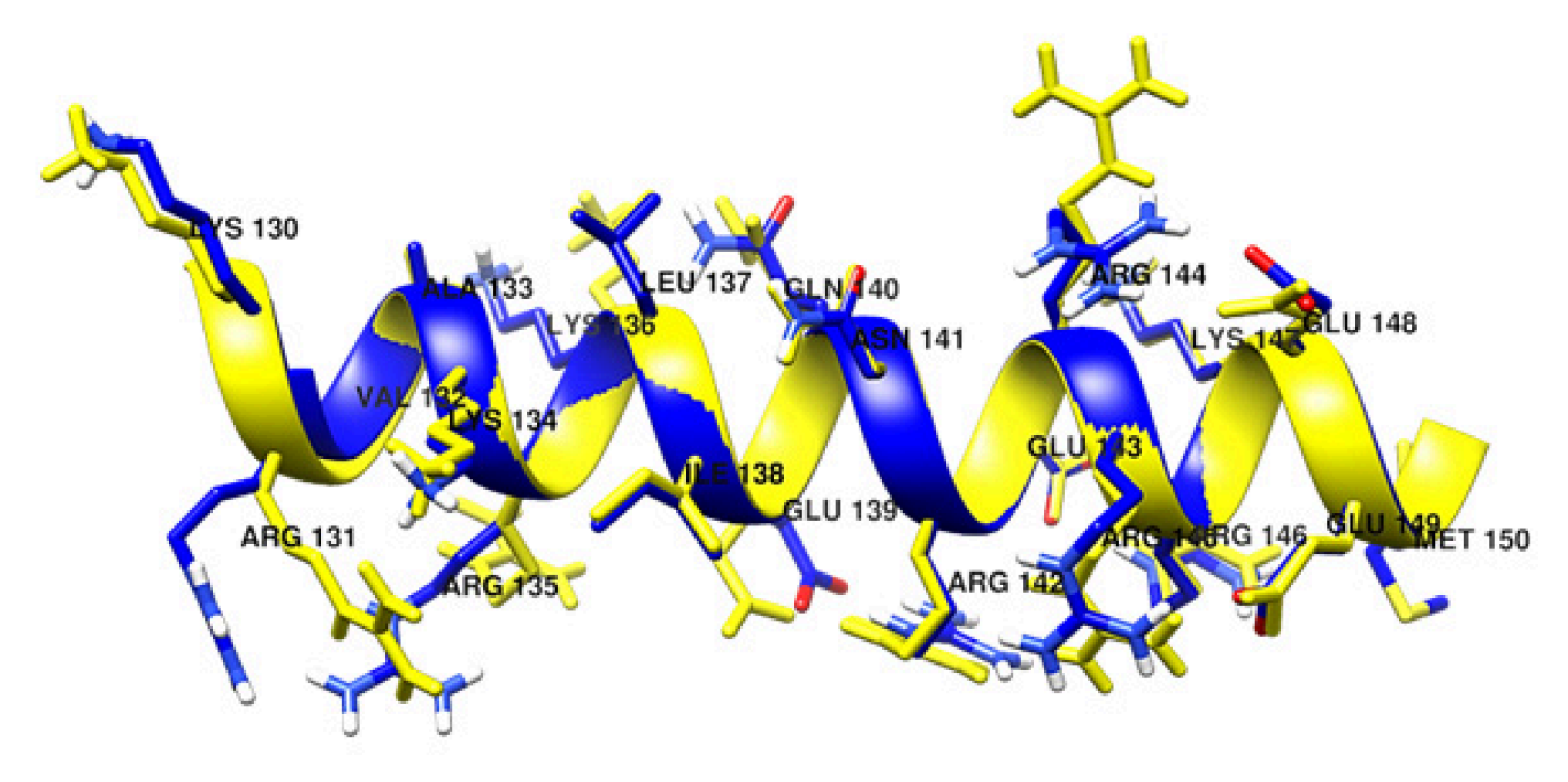
Aurantinidin	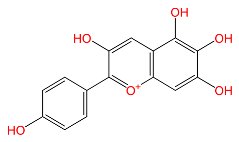	−3885.28	2.578	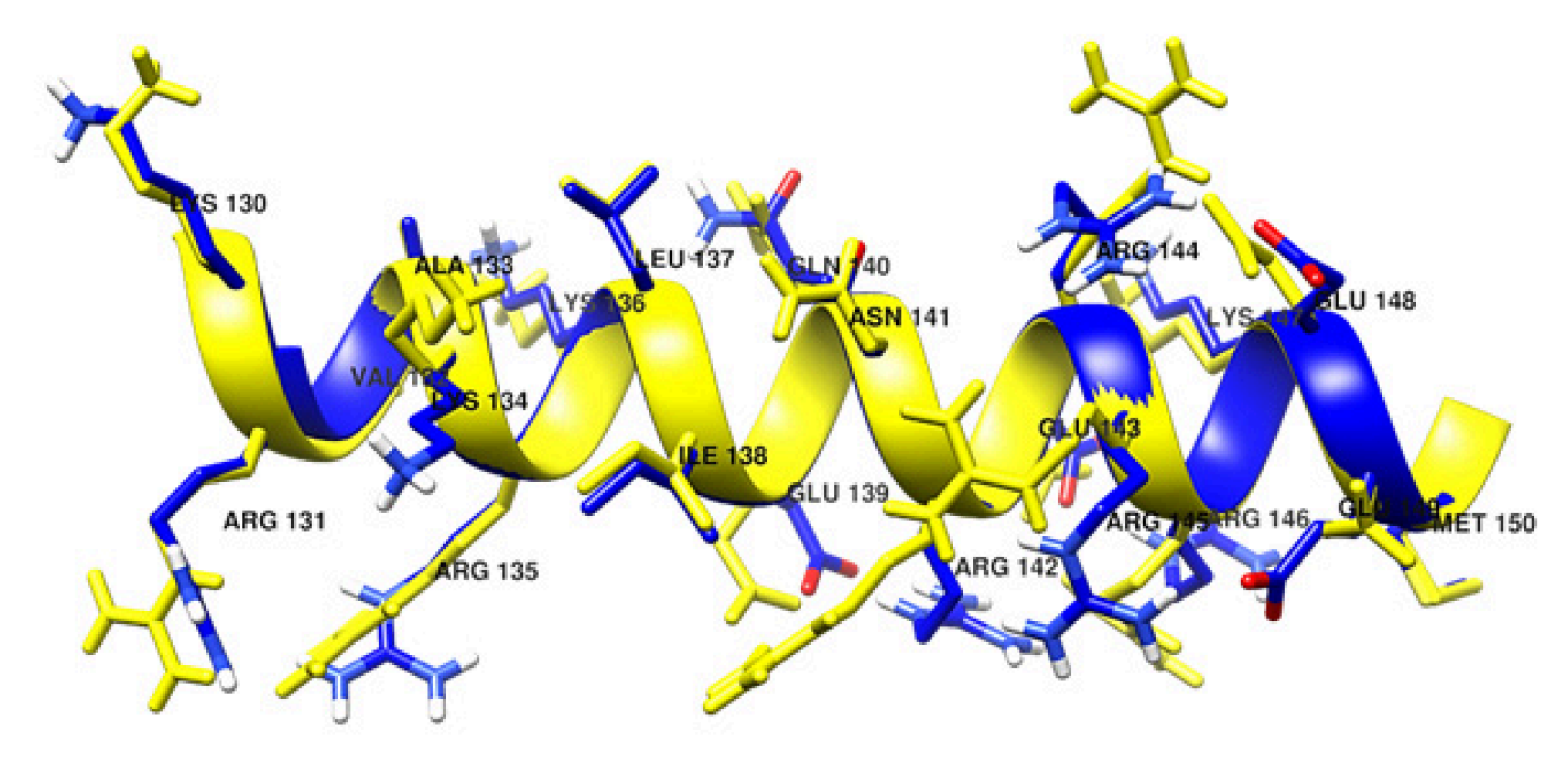
Caffeic Acid	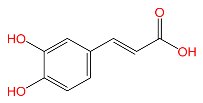	−4120.37	1.839	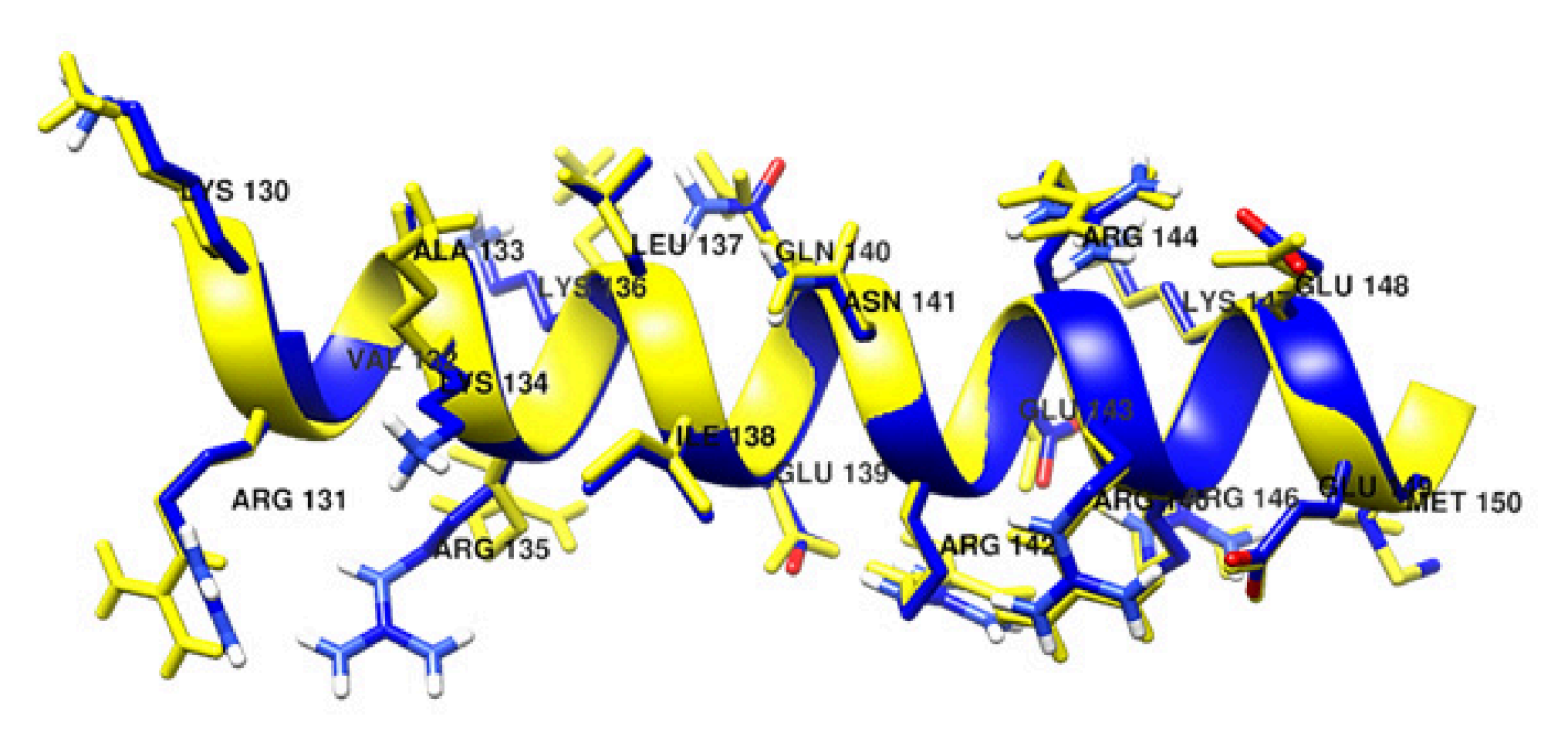
Caffeic Acid Phenethyl Ester	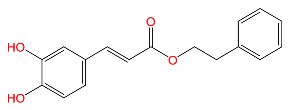	−4426.43	2.715	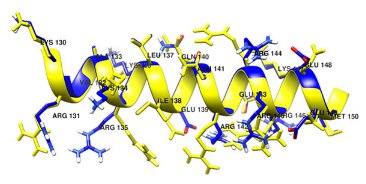
Chlorogenic Acid	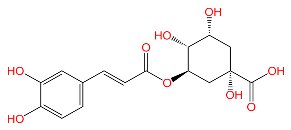	−4669.35	2.919	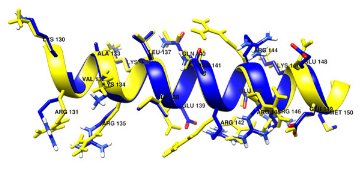
Chlorogenic Acid Methyl Ester	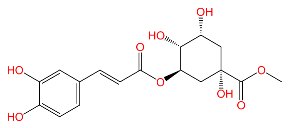	−3965.88	1.687	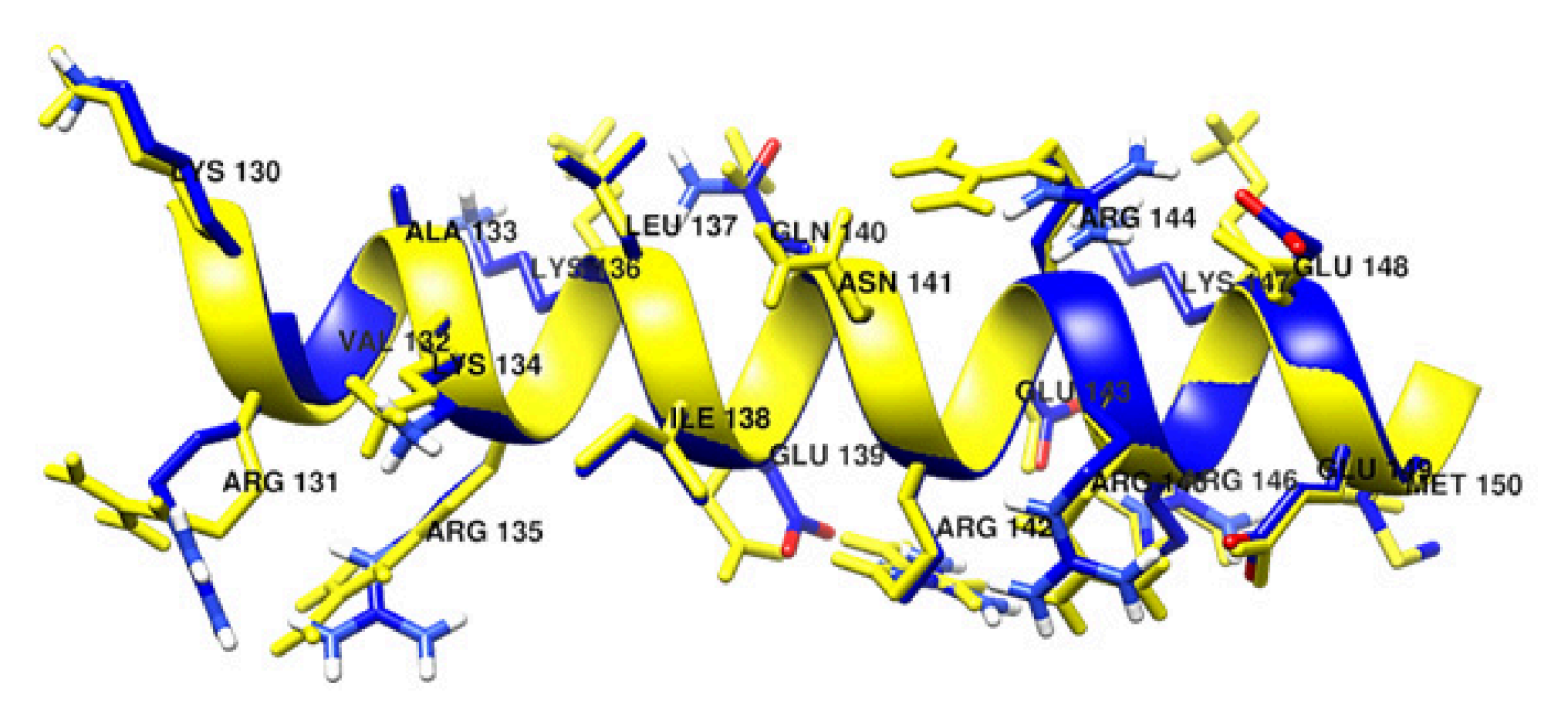
p-Coumaric Acid	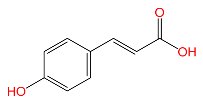	−4346.56	2.619	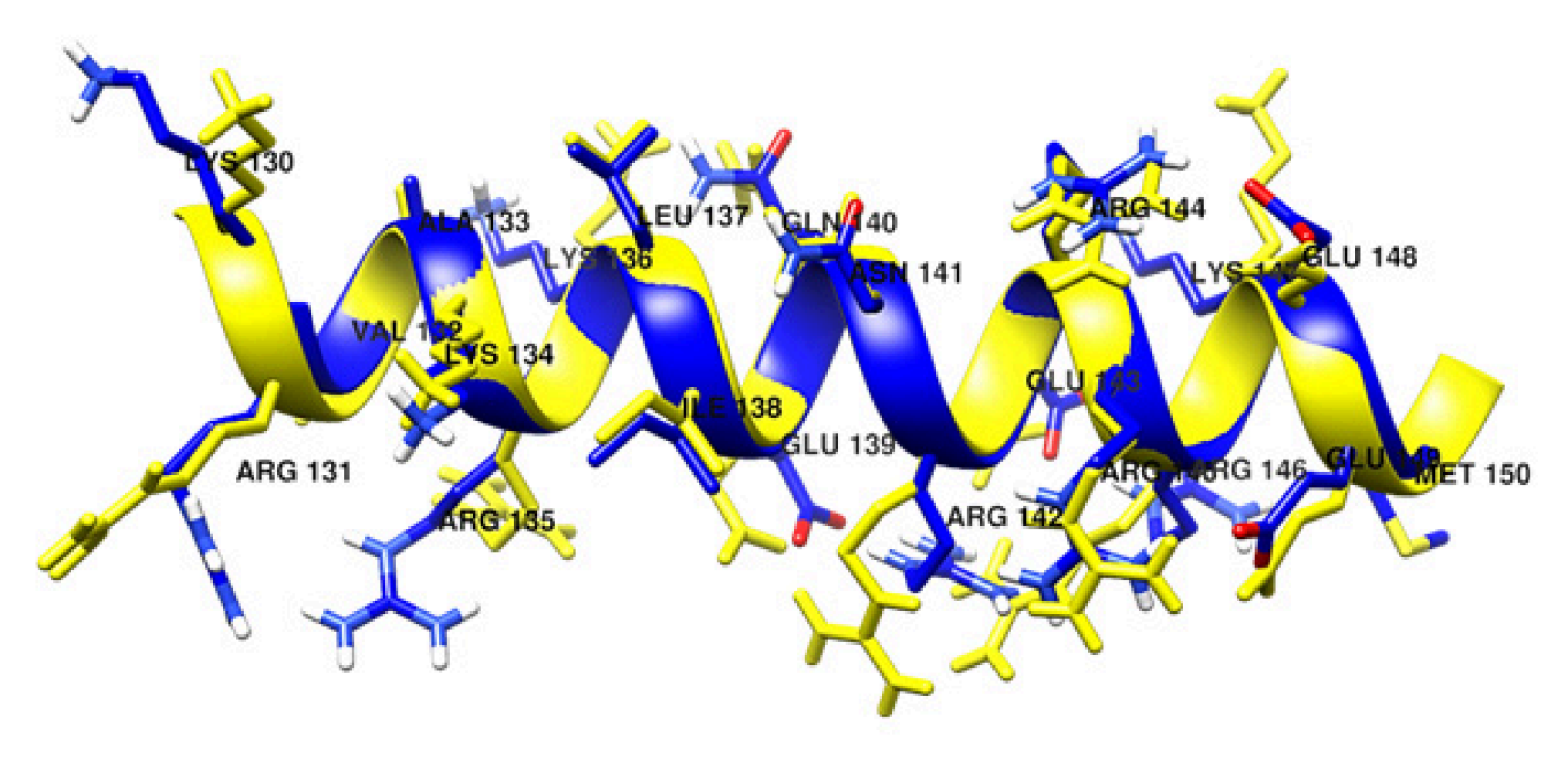
Curcumin	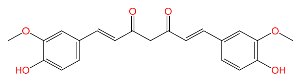	−3735.04	2.464	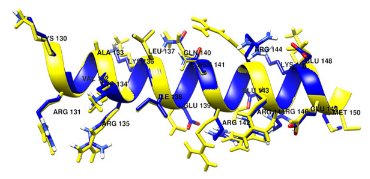
Epicatechin	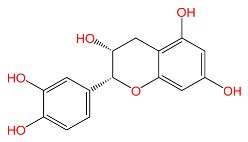	−3263.79	1.709	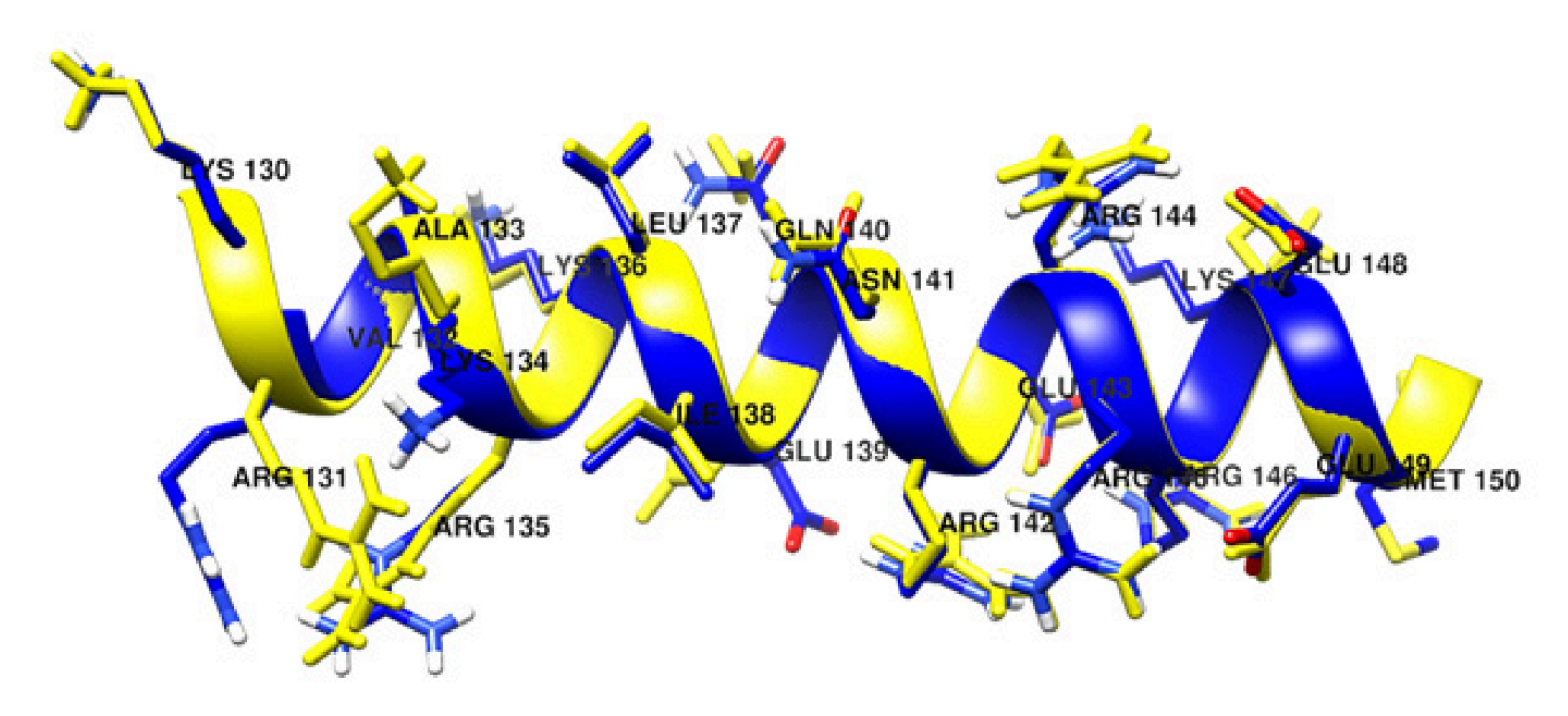
Ferulic Acid	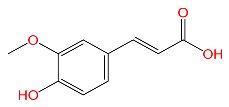	−3588.84	1.393	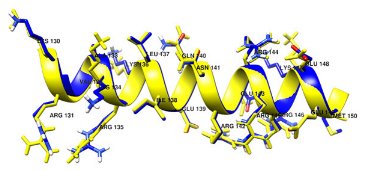
Genistein	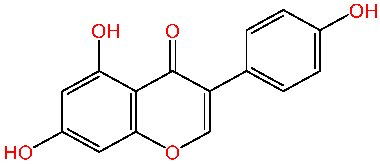	−3446.47	2.026	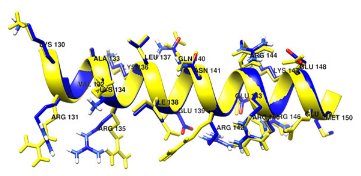
Methyl-4-Hydroxycinnamate	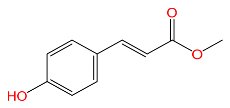	−3888.82	2.044	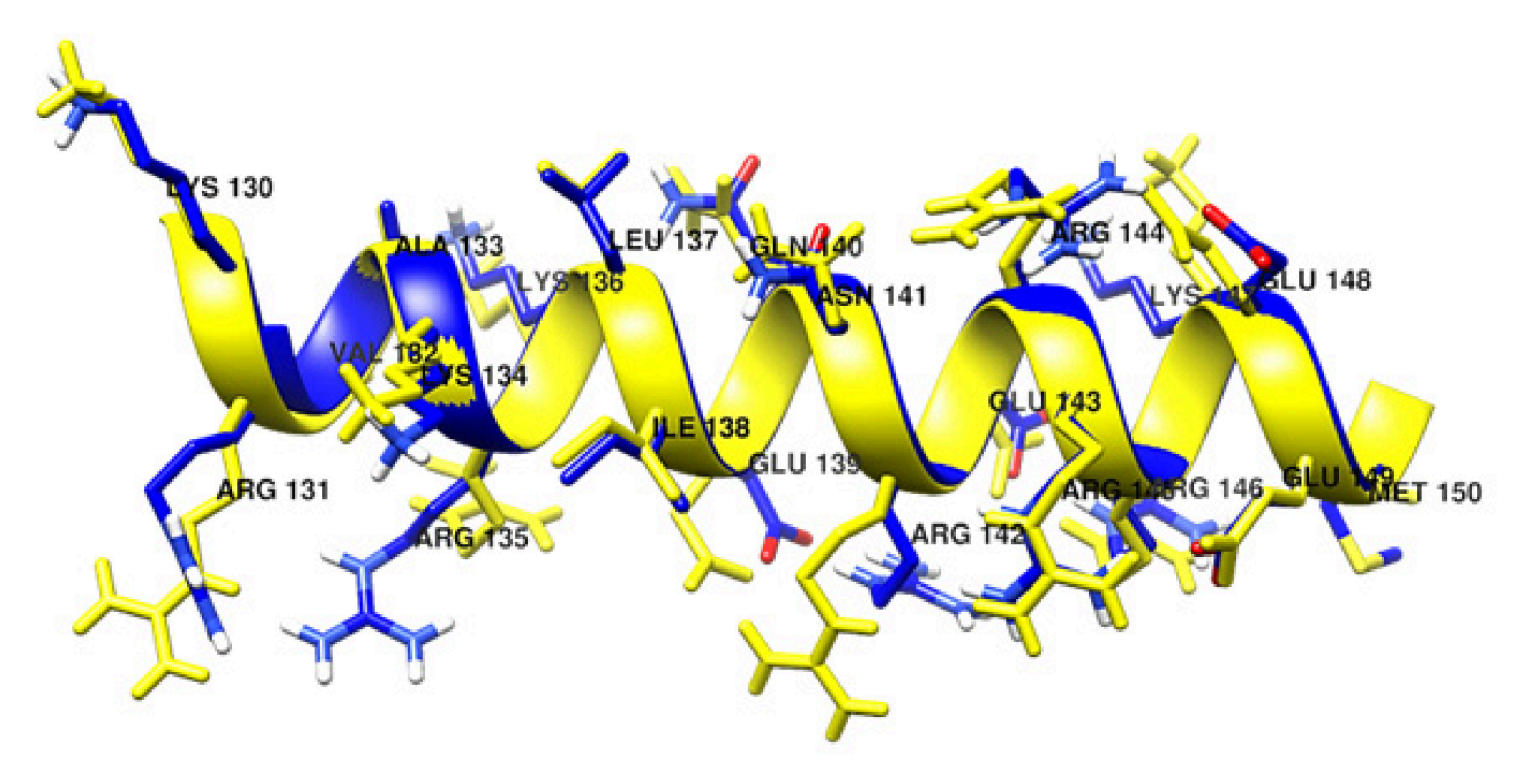
O-Methylsinapate	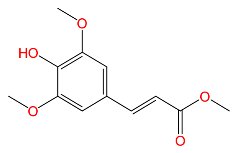	−4126.83	2.552	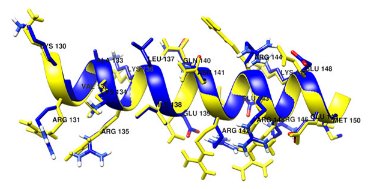
P1 *	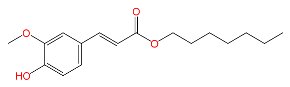	−4202.06	2.293	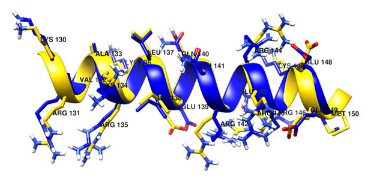
P2 *	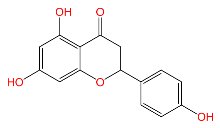	−3790.83	2.499	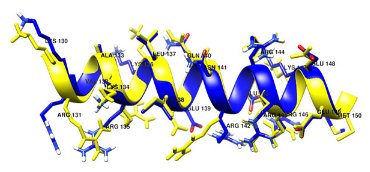
P3 *	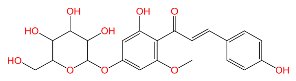	−3302.65	2.082	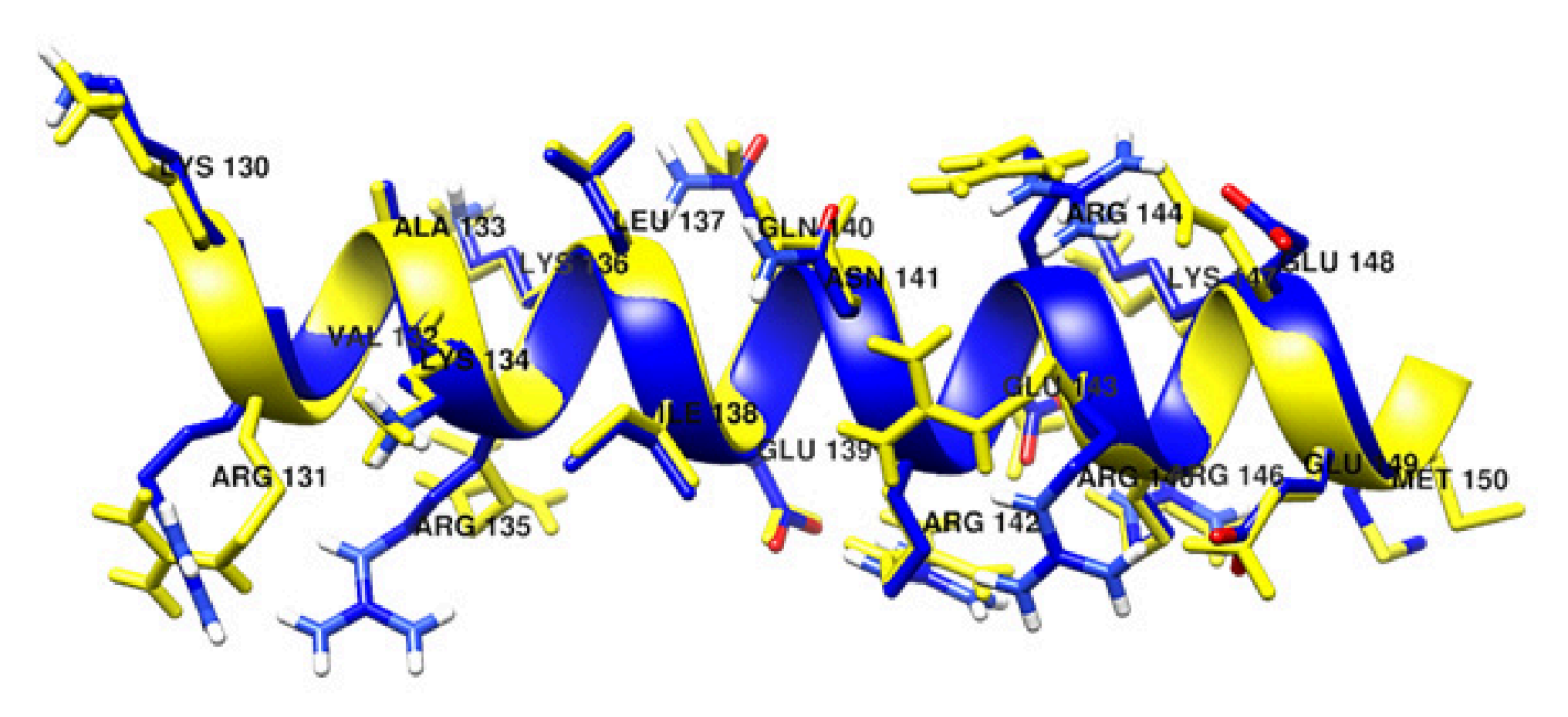
P4 *	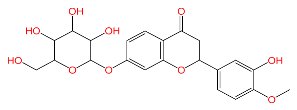	−5171.51	2.536	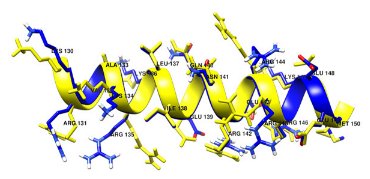
Quercetin-3-Galactoside	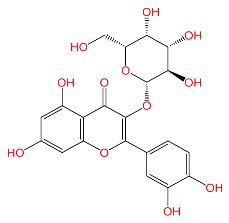	−3180.92	2.523	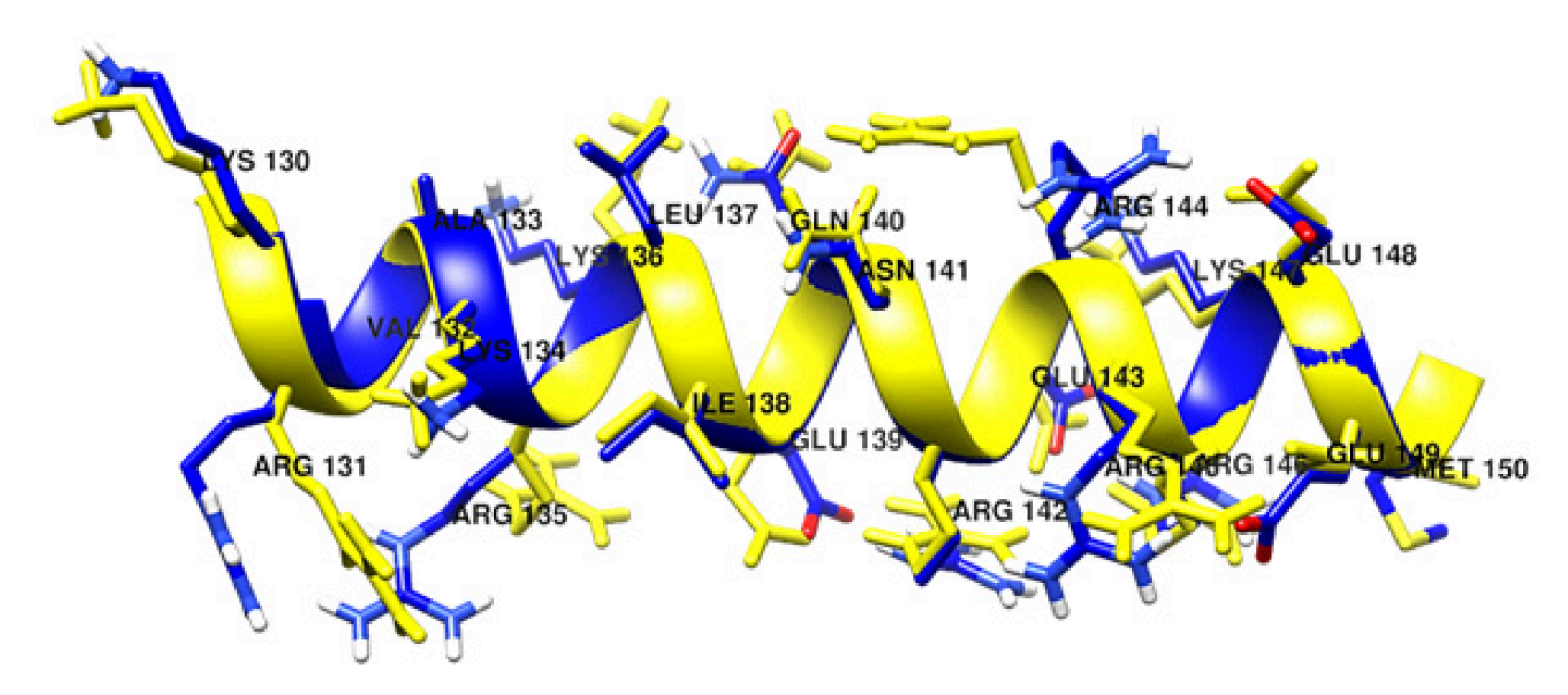
Quercetin-3-Glucoside	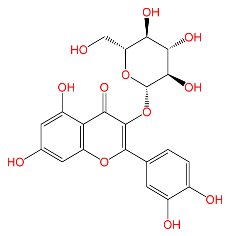	−4400.19	2.424	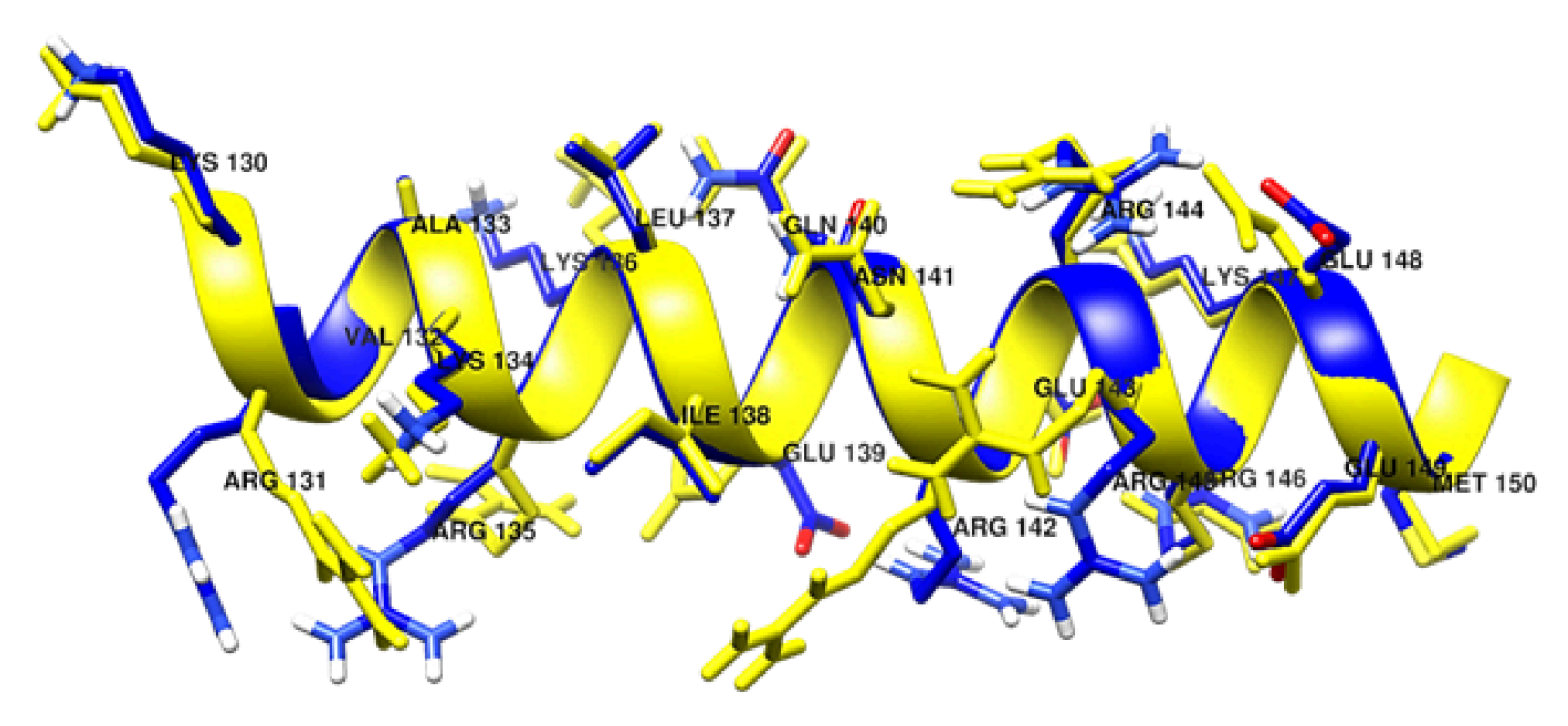
Sinapic Acid	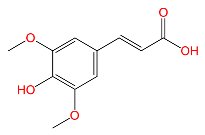	−3975.09	1.798	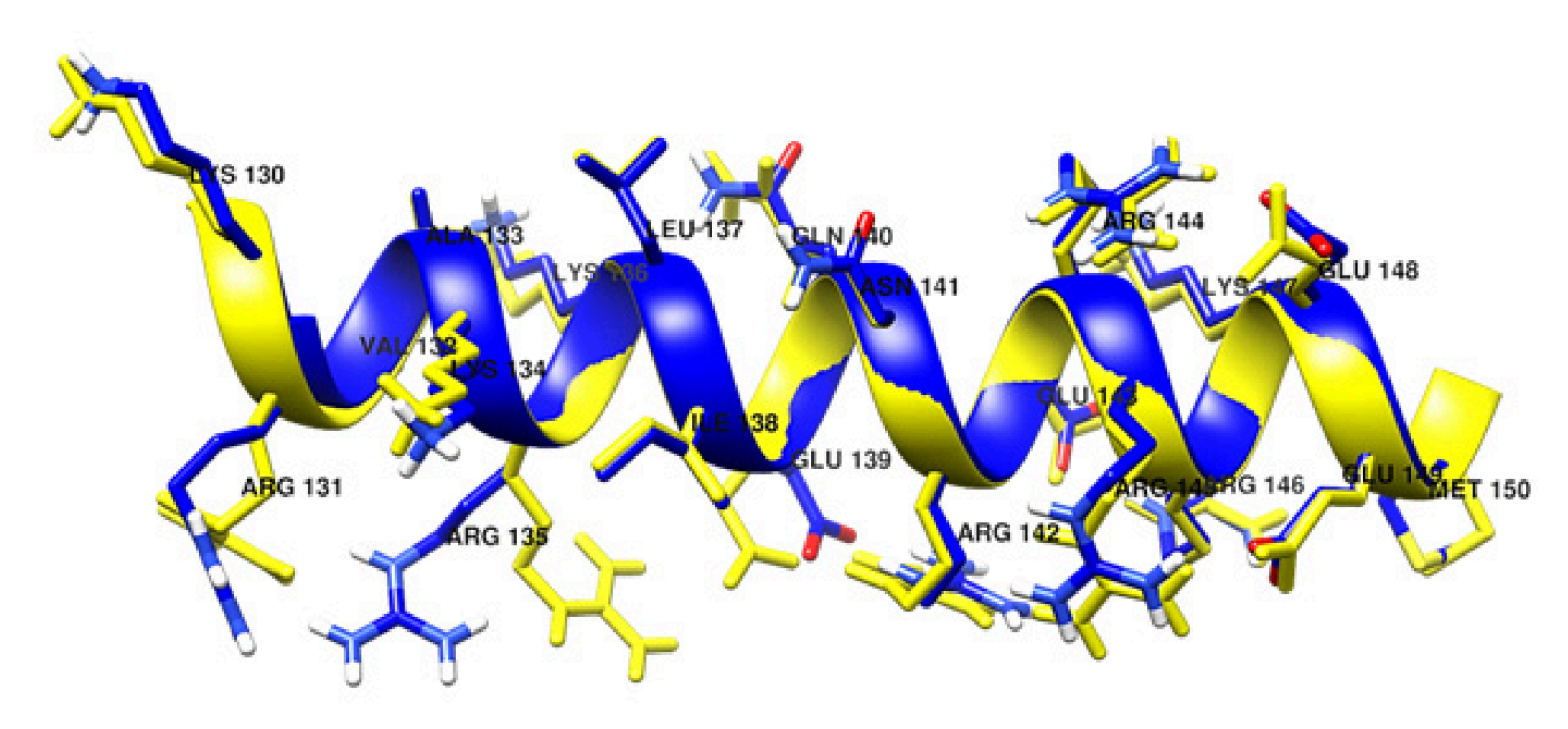

## Data Availability

The original contributions presented in this study are included in the article. Further inquiries can be directed to the corresponding authors.
